# Split & mix assembly of DNA libraries for ultrahigh throughput on-bead screening of functional proteins

**DOI:** 10.1093/nar/gkaa270

**Published:** 2020-05-08

**Authors:** Laurens Lindenburg, Tuomas Huovinen, Kayleigh van de Wiel, Michael Herger, Michael R Snaith, Florian Hollfelder

**Affiliations:** 1 Department of Biochemistry, University of Cambridge, 80 Tennis Court Rd, Cambridge CB2 1GA, UK; 2 AstraZeneca Medimmune Cambridge, Antibody Discovery and Protein Engineering, Cambridge, UK

## Abstract

Site-saturation libraries reduce protein screening effort in directed evolution campaigns by focusing on a limited number of rationally chosen residues. However, uneven library synthesis efficiency leads to amino acid bias, remedied at high cost by expensive custom synthesis of oligonucleotides, or through use of proprietary library synthesis platforms. To address these shortcomings, we have devised a method where DNA libraries are constructed on the surface of microbeads by ligating dsDNA fragments onto growing, surface-immobilised DNA, in iterative split-and-mix cycles. This method—termed SpliMLiB for **Spli**t-and-**M**ix **Li**brary on **B**eads—was applied towards the directed evolution of an anti-IgE Affibody (Z_IgE_), generating a 160,000-membered, 4-site, saturation library on the surface of 8 million monoclonal beads. Deep sequencing confirmed excellent library balance (5.1% ± 0.77 per amino acid) and coverage (99.3%). As SpliMLiB beads are monoclonal, they were amenable to direct functional screening in water-in-oil emulsion droplets with cell-free expression. A FACS-based sorting of the library beads allowed recovery of hits improved in *K*_d_ over wild-type Z_IgE_ by up to 3.5-fold, while a consensus mutant of the best hits provided a 10-fold improvement. With SpliMLiB, directed evolution workflows are accelerated by integrating high-quality DNA library generation with an ultra-high throughput protein screening platform.

## INTRODUCTION

Site-selective combinatorial DNA libraries increase the efficiency of protein screening campaigns by focusing on the randomisation of amino acids most likely to yield improvements (1). The design of such ‘smart’ libraries, targeting one or more pre-selected positions has been greatly facilitated by mechanistic and structural insight (2,3) and can outperform random mutagenesis methods (4). Targeting each additional site for saturation increases library size exponentially so that, unless the amino acid ‘alphabet’ is reduced to keep variant numbers manageable (5), highly efficient screening assays are called for. The effective diversity of the library is reduced, if library members are duplicated, e.g. due to biased introduction of nucleotides leading to redundancy. Maximal diversity is achieved in *balanced* libraries in which ideally each alternative codon is represented in equal measure, so that none of the potentially beneficial mutations introduced in the ‘smart’ library design are missed during screening.

A common method for creating combinatorial libraries is to use oligonucleotides that introduce codons synthesised as mixed bases (e.g. NNK) (6–8). Such oligonucleotides are relatively inexpensive and multiple mixed-based codons can be combined on the same oligonucleotide but the quality of DNA libraries is compromised as they introduce degeneracy and encode unequal proportions of amino acids (9). The degeneracy problem has been partially addressed through the development of ‘small-intelligent libraries’, using a blend of different mixed-base codon-containing oligonucleotides (e.g. ‘22c-trick’), although such approaches cannot deliver custom codon ratios and the targeting of multiple sites in close proximity is still challenging (10,11). TRIM technology, where defined blocks of nucleotide trimers are incorporated during phosphoramidite synthesis, enables full control over codon balance but remains relatively expensive (12–14). Furthermore, robotic techniques such as Slonomics and Colibra have been developed to deliver highly customised 3-nucleotide additions (using ligation), but these techniques remain essentially proprietary and inaccessible to the wider research community (15,16).

The use of site saturation libraries generally entails a cellular transformation step, implying a potential bottlenecking of the population, unless significant resources (in the form of labour or capital) are allocated to transforming a sufficiently large number of cells. Furthermore, without a suitable ultra-high throughput assay to screen the transformants, only a limited fraction of the total library size might be practically accessible (17). Seminal work by Griffiths and Tawfik first demonstrated the use of emulsion droplets in enzyme evolution, where proteins were expressed from single molecules of DNA in droplets containing *in vitro* transcription/translation (IVTT) mixture (18). Protein expression from a single DNA molecule in the droplet guarantees the correct genotype-phenotype linkage in a ‘monoclonal droplet’. The use of microbeads with moieties to pull-down expressed proteins within droplets has further aided selection schemes, by allowing many monoclonal protein copies to be interrogated simultaneously using well-established flow-cytometry-based sorting, improving signal-to-noise ratio in the assay (19,20). Furthermore, beads have allowed separation of the mutually incompatible DNA amplification and cell-free expression reactions, typically by use of an initial emulsion PCR step (21–27). Despite these latter examples, several difficulties remain with the DNA amplification step and beads: (i) the Poisson distribution dictates that ∼80% of beads be left not carrying any DNA if the majority of beads that do carry DNA are to be monoclonal; (ii) emulsion PCR has been found to steadily decrease in yield with increasing length of template (25); iii) the high temperature of PCR conditions places stringent demands on the DNA surface attachment chemistry (28).

We sought therefore to develop a fully non-degenerate site-saturation mutagenesis method that would be user-friendly (by avoiding the need for robotics, specialist reagents or multiple PCR work-up steps), free of cellular transformations (to maintain maximal library diversity) and interfacing directly with ultrahigh throughput screens in the powerful format of emulsion microdroplets (29). We devised a DNA assembly method based on ligation of oligonucleotide duplexes directly on a microbead surface, resulting in a ‘one-bead-one-protein’ library in which every bead of the library is densely coated in DNA, representing a single ‘genotype’ and encoding a single protein-of-interest (PoI) variant. Combinatorial diversity of the ligated fragments is introduced by a split & mix approach, reminiscent of the peptide synthesis scheme first employed by Knapp and co-workers, who pioneered the ‘one bead, one compound’ approach (30) as well as by ‘encoded combinatorial chemistry’, where chemical steps are encoded through linked DNA modifications, invented by Brenner (31). SpliMLiB (**Spli**t-and-**M**ix **Li**brary on **B**eads) was directly applied to screening for protein-binder functionality, by compartmentalising single beads into the droplets of a polydisperse water-in-oil emulsion, together with IVTT mix. Flow cytometric sorting of these display beads after incubation with a fluorescently labelled antigen led to successful isolation of protein binders, Affibody molecules with enhanced affinity.

## MATERIALS AND METHODS

### General paramagnetic bead handling

Tween-20 was *always* included at 0.02 - 0.05% (v/v) in *all* solutions coming into contact with paramagnetic beads. This applies both to beads used for solid-phase library build-up and protein display and beads in the slurry used for SPRI-based DNA purification. It includes all enzymatic reactions (ligations and restrictions). In absence of Tween-20, severe bead clumping and a detrimental effect on results was noticed. The only exceptions were the IVTT reaction, and the KBBK bind & wash buffers (see below for details): these solutions were not supplemented with Tween-20 as they were not found to cause clumping in absence of supplemental Tween-20. Beads were washed with phosphate buffered saline with Tween-20 (PBST, consisting of 8 mM Na_2_HPO_4_, 150 mM NaCl, 2 mM KH_2_PO_4_, 3 mM KCl, 0.05% (v/v) Tween-20, pH 7.4). Supernatant was aspirated while magnetically fixing beads in 1.5–2 ml-sized Eppendorf tubes on a bar magnet (DynaMag-2 Magnet, ThermoFisher Scientific) or in 0.2 ml PCR tubes on a 96-well magnet (DynaMag-96 Side Magnet, ThermoFisher Scientific). Beads were routinely counted using disposable cell-counting chambers and a transmitted light microscope.

### Preparation of beads with modified surface for DNA library build-up and protein display

Tamavidin-2-HOT-SpyTag was covalently coupled to paramagnetic carboxy beads (Ø 5 μm; S1964, microParticles, Berlin). Beads (100 mg) were washed with water, then resuspended in 1 ml water. To the bead suspension was added 0.5 ml of 750 mM of *N*-(3-dimethylaminopropyl)-*N*′-ethylcarbodiimide hydrochloride (EDC, Sigma-Aldrich, **1** in Supplementary Figure S1A) in water with 0.02% (v/v) Tween-20 and the mixture was incubated for 20 min. The supernatant was removed, the beads were washed once with water (with 0.05% (v/v) Tween-20), before they were resuspended in 5 ml of 25 mM sodium phosphate buffer (pH 5.8), with 0.05% (v/v) Tween-20. Subsequently, Tamavidin-2-HOT-SpyTag fusion protein (1.5 ml of 10 mg/ml in PBST) was added and the tube was left on a roller at room temperature for four hours. Finally, the beads were washed with and incubated for 10 min in 0.5 M Tris–HCl (pH 8), followed by washing with PBST. To functionalise the coupled protein with Azido-PEG4-NHS, 100 mg of Tamavidin-SpyTag-coupled beads in 400 μl of PBST was mixed with 400 μl of Azido-PEG4-NHS ester (50 mM in DMSO, Jena Biosciences, **2** in Supplementary Figure S1B), for a final 50% (v/v) DMSO concentration. The beads were incubated at room temperature for 2 h with vigorous shaking, followed by washing with PBST. Successful azido functionalisation was tested for as set out in Supplementary Figure S1C&D. SpyTag functionality was confirmed as set out in Figure S1E. Beads incubated with GFP-SpyCatcher displayed a median fluorescence in flow cytometry that was 400-fold higher than observed with beads incubated with GFP, indicating dense, functional coating of SpyTag on the beads.

### Oligonucleotides used in this study

Commercially obtained oligonucleotide sequences, 5′-modifications, synthesis scales and purification method are set out, both for common oligonucleotides used in this study (Supplementary Table S1) and for variation-encoding oligonucleotides used for the Z_IgE_ SpliMLiB library (Supplementary Table S2), with codons used for site saturation indicated separately (Supplementary Table S3).

### Molecular cloning of individual constructs and of selected hits from screening

Z_IgE_^wild-type^ and Z_IgE_^nonbinder-1^ were synthesised as DNA fragments (GeneArt Strings, ThermoFisher Scientific) and cloned into a modified pIVEX-2.3d vector (biotechrabbit GmbH) that carried a SpyCatcher-encoding sequence, resulting in vectors pIVEX-Z_IgE_^wild-type^-SpyCatcher and pIVEX-Z_IgE_^nonbinder-1^-SpyCatcher (Supplementary Figure S2A, B). To generate the construct pIVEX-CaBoFDH, a synthetic DNA fragment was ordered (GeneArt Strings, ThermoFisher Scientific), restricted with NdeI and NotI and ligated into a modified version of pIVEX2.4d that had been digested with the same restriction enzymes (Supplementary Figure S2C). To allow bacterial expression of the Z_IgE_-SpyCatcher constructs, the pIVEX-Z_IgE_^wild-type^-SpyCatcher and pIVEX-Z_IgE_^nonbinder-1^-SpyCatcher vectors were restricted with NdeI and BamHI and the resulting inserts were ligated into pET28a cut with the same restriction enzymes, resulting in the constructs pET28a-Z_IgE_^wild-type^-SpyCatcher and pET28a-Z_IgE_^nonbinder-1^-SpyCatcher, containing both an N- and a C-terminal His-tag (Supplementary Figure S2D). To recover DNA after FACS selection of beads, PCR reactions (conditions as described below for SpliMLiB input fragments) were performed using the sorted beads as template and with primers SfiI_F and SfiI_R (Supplementary Table S1). The PCR reactions were purified using the Solid Phase Reversible Immobilisation (SPRI) bead protocol (as described below) and subsequently 1 μg of amplicon was treated with 10 units of SfiI restriction enzyme (ThermoFisher) in a 20 μl reaction at 50°C. The restriction reactions were purified over silica columns (Clean & Concentrate, Zymo, Irvine, CA, USA) and ligated into pET28a-Z_IgE_^nonbinder-1^-SpyCatcher also cut with SfiI. This ensured the 223 bp amplicon incorporating all four targeted mutational saturation sites in the library was subcloned into the bacterial expression vector. The individual constructs pET28a-Z_IgE_^nonbinder-2^-SpyCatcher and pET28a-Z_IgE_^consensus^-SpyCatcher were generated from separately assembled solid-phase ligation fragments, omitting the splitting steps, but using instead only the appropriate fragments, following the protocols described below. The fragments were SfiI digested, allowing ligation with the backbone from SfiI-digested pET28a-Z_IgE_^nonbinder-1^-SpyCatcher. For *K*_d_ determination by biolayer interferometry, several Z_IgE_-SpyCatcher variants were furnished with an N-terminal Avi-tag fusion for site-specific biotinylation. The plasmids pET28a-Z_IgE_^wild-type^-SpyCatcher and pET28a-Z_IgE_^consensus^- SpyCatcher were digested with NcoI and NotI and the resulting fragments were ligated into a derivative of a pHAT vector with an N-terminal Avi tag that had been digested with the same restriction enzymes, resulting in pHAT-Avi- Z_IgE_^wild-type^-SpyCatcher (see Supplementary Figure S2E for partial plasmid DNA sequence) and pHAT-Avi-Z_IgE_^consensus^-SpyCatcher. To generate pHAT-Avi- Z_IgE_^nonbinder-2^-SpyCatcher, pHAT-Avi- Z_IgE_^33^-SpyCatcher and pHAT-Avi-Z_IgE_^44^-SpyCatcher, NcoI/NotI restriction fragments from pET28a-Z_IgE_^nonbinder-2^-SpyCatcher, pET28a-Z_IgE_^33^-SpyCatcher and pET28a-Z_IgE_^44^-SpyCatcher, respectively, were ligated into a backbone generated through digestion of pHAT-Avi- Z_IgE_^consensus^-SpyCatcher with NcoI and NotI. The construct pET28a-Tamavidin-2-HOT-SpyTag is described elsewhere (Huovinen *et al.* 2020, to be submitted).

### Bacterial protein expression & purification

Expression of pET28a-based constructs was carried out in volumes of 250 ml (pET28a-Z_IgE_-SpyCatcher) to 0.5 L (pET28a-Tamavidin-2-HOT-SpyTag) LB culture media (containing 50 μg/ml kanamycin). These were started using *E. coli* BL21(DE3) colonies scraped from agar plate. *E. coli* were grown in a shaking incubator to an OD_600_ of 0.5 at 37°C, 100 μM IPTG was added and the cultures grown further overnight at 25°C. For the expression of pHAT-Avi-based constructs, BirA-expressing *E. coli* BL21(DE3) (250 ml) of the pHAT-Avi-Z_IgE_-SpyCatcher constructs were inoculated through the scraping of bacterial colonies from agar plates. LB cultures (with 100 μg/ml carbenicillin and 20 μg/ml chloramphenicol) were grown to an OD_600_ of 0.5 at which time the cultures were induced through the addition of 100 μM IPTG, while 40 μg/ml biotin was added to allow for BirA to catalyse the addition of biotin to the lysine in the BirA tag. Cultures were then incubated overnight at 25°C. Following protein over-expression, cells were pelleted, lysed with 10 ml of BugBuster with 2500 units of Benzonase endonuclease (Novagen) before the lysate was clarified by centrifugation and applied to a Ni-NTA gravity flow column (1 ml bed volume, Ni-NTA agarose, Qiagen). The column was washed with 20 column volumes of wash buffer (20 mM Tris–HCl (pH 8), 500 mM NaCl, 30 mM imidazole) and eluted with elution buffer (20 mM Tris–HCl (pH 8), 500 mM NaCl, 500 mM imidazole). The eluate was concentrated by centrifugation through tubes containing filters with molecular weight cut offs (MWCO) of 3 kDa (Z_IgE_-SpyCatcher constructs) or 10 kDa (Tamavidin-2-HOT-SpyTag), before being desalted using PD-10 columns (GE) equilibrated with PBS. Both Tamavidin-2-HOT-SpyTag and Z_IgE_-SpyCatcher variants were obtained in good yield (both ∼80 mg/L LB) and purity.

### PCR fragment generation

PCR fragments were required for SpliMLiB, both for the optimisation experiments set out in Figure 2 and the preparation of fragments for the Z_IgE_ library. PCR fragments were prepared using 500 μl reactions consisting of 0.5 μM of each forward and reverse primer, 1× BIOTAQ NH4 buffer, 3 mM MgCl_2_, 1 mM dNTPs, 0.5 ng/μl plasmid template and 0.05 units/μl BIOTAQ DNA polymerase (BIOTAQ polymerase and buffer were from Bioline, London, England). Reaction setup (in terms of primers & template) is set out in Supplementary Table S4. Thermocycling was performed starting with 2 min at 96°C, followed by 30 cycles of 15 s at 96°C, 15 s at 55°C, 45 s at 72°C, followed by a final extension step at 72°C for 1 min.

### Solid phase reversible immobilisation (SPRI)-based purification of PCR reactions

PCR reactions were purified by SPRI beads (32). The SPRI slurry was prepared with 1 ml of 50 mg/ml bead stock (SpeedBeads magnetic carboxylate modified particles, 1 μm ø, GE Healthcare), suspended in a 49 ml solution of 20% (w/v) PEG-8000, 2.5 M NaCl, 0.05% (v/v) Tween-20. One volume of PCR reaction was mixed with two volumes of SPRI slurry, incubated for 5 min, before the supernatant was removed on a magnet stand. While keeping on the magnet stand, the beads were washed twice with 70% (v/v) ethanol and 0.05% (v/v) Tween-20. Elution of DNA from the SPRI beads was carried out with water with 0.02% (v/v) Tween-20.

### PCR fragment restriction in solution

PCR fragments that were to be ligated to bead-immobilised DNA, required cohesive ends. For the assembly set out in Figure 2C, a 5′-overhang in PCR product ‘frag_1_’ (Supplementary Table S4) was introduced by restriction with BspQI: a 30 μl reaction consisting of 150 pM DNA, 1× buffer 3.1 (NEB) and 30 units of BspQI (NEB), was incubated at 50°C for 2 h, followed by inactivation of the restriction enzyme by heating to 80°C for 20 min. 5′-Overhangs in frag_T10_ PCR fragments for the final fragment ligation in the Z_IgE_ SpliMLiB library (Figure 3C, step viii) were introduced by restriction with Esp3I, in 50 μl reactions consisting of 70–100 pM of purified PCR fragment, 50 units of Esp3I (ThermoFisher Scientific), 1× buffer Tango (ThermoFisher Scientific) supplemented with 1 mM DTT. The restriction reactions were incubated at 37°C for 2 h followed by 20 min at 65°C to heat-inactivate Esp3I. In both cases, the restricted DNA was purified using the SPRI bead protocol described above.

### Generation of oligonucleotide duplex fragments and their enzymatic 5′-phosphorylation

In SpliMLiB, bead surface-bound DNA was occasionally extended with pairs of hybridised oligonucleotides (e.g. as set out in steps iv and vi in Figure 3C). Oligonucleotide pairs used to generate the duplexes are set out in Supplementary Table S5. Oligonucleotides were first enzymatically phosphorylated at their 5′-ends in separate 30 μl reactions consisting of 450 pmol oligonucleotide, 15 units of T4 polynucleotide kinase (NEB), 1× T4 DNA ligase reaction buffer (NEB), that were incubated at 37°C for 30 min, followed by heat inactivation of the kinase at 65°C for 20 min. To hybridise complementary oligonucleotide pairs, the phosphorylated oligonucleotides were mixed at 25 μl each, then subjected to heating for 2 min at 95°C, followed by 10 min at 52°C and a final cooling down to 4°C. These duplexes were used for solid-phase ligation without further purification.

### Covalent coupling of DNA to bead surface

To effect covalent immobilisation of either full-length constructs or the set of 20 initial SpliMLiB fragments on Tamavidin-SpyTag and azido-functionalised paramagnetic microbeads (e.g. for step i in Figure 3C), the Dynabeads kilobaseBINDER Kit (KBBK, ThermoFisher Scientific) was used. This kit is designed to enhance the efficiency of immobilisation of biotinylated DNA on streptavidin beads through provision of molecular crowding conditions and we found it to equally enhance the efficiency of the copper-free click reaction between DBCO on DNA and azide on bead. Tamavidin-SpyTag and azide functionalised paramagnetic microbeads were washed once in 40 μl of the Binding Solution from the KBBK, then resuspended in a mixture of 40 μl of Binding Solution and 40 μl of DBCO-functionalised DNA fragment in water. DNA was added at a ratio of at least 20 million copies DNA per bead, while reactions contained 1–3 million beads in total. The beads were incubated at 37°C with shaking at 1200 RPM for 1 h, after which supernatant was removed on the bar magnet, the beads were washed once with 40 μl KBBK Wash Solution and then washed three times with PBST. We found that providing 20 million copies (as determined by absorbance spectroscopy) of fluorescein-conjugated DNA per bead resulted in a readily detectable fluorescent signal with flow cytometry (Supplementary Figure S1D); adding fewer DNA molecules made following the efficiency of subsequent reactions difficult (not shown).

### Solid-phase DNA restriction

To restrict bead-surface-immobilised DNA, beads were washed once in 1× Tango restriction buffer (ThermoFisher Scientific) supplemented with 1 mM DTT and 0.02% (v/v) Tween-20 (for Esp3I) or in 1× Buffer 3.1 with 0.02% (v/v) Tween-20 (for BspQI). To effect Esp3I-digestion (e.g. for step iii in Figure 3C), beads were then incubated for 2 h at 37°C, while shaking at 1200 RPM, in a solution of 200 units of Esp3I, 1 mM DTT, in 1× Tango buffer (ThermoFisher Scientific, in a total volume of 120 μl. Alternatively, to effect BspQI-digestion (i.e. for restriction after ligation of Frag_2_ in Figure 2C), beads were incubated for 2 h at 50°C in a non-shaking thermocycler with heated lid, in a solution of 40 units of BspQI, in 1× Buffer 3.1 (NEB), in a total volume of 120 μl. Both digestions were followed by the three washes with PBST.

### Solid-phase DNA ligation

Ligation of soluble DNA to bead-bound DNA is integral to the SpliMLiB method (e.g. see steps iv, vi and viii in Figure 3C). To prepare beads for ligation of an incoming DNA fragment, the beads were washed once in 1× T4 DNA ligase reaction buffer (NEB), supplemented with 0.02% (v/v) Tween-20. To ligate oligonucleotide duplex fragments, beads (1 million per tube split) were incubated with 45 μl phosphorylated oligonucleotide duplex (338 pmol), 5.5 μl 10× T4 DNA ligase reaction buffer (NEB), 5 μl T4 DNA ligase (2000 units, NEB), 4.5 μl 100 mM DTT, 1.5 μl 30 mM ATP, 38.5 μl water with 0.02% (v/v) Tween-20. This reaction setup accounted for salts contributed by the unpurified phosphorylated oligonucleotide duplex and supplemented it with possibly depleted ATP and DTT. Beads were incubated at 16°C for 1 h and then washed three times with PBST. To ligate incoming, solution-phase, Esp3I or BspQI-treated PCR fragments to DNA with cohesive end immobilised on beads, the beads (1 million per tube split) were incubated with 35–50 pmol of DNA fragment, 1× T4 DNA ligase reaction buffer and 1200 units of T4 DNA ligase in a total reaction volume of 50 μl, with 0.02% (v/v) Tween-20. Beads were incubated at 16°C for 1 h and then washed three times with PBST.

### Next generation sequencing by Illumina MiSeq

NGS sequencing of the 160 000-member SpliMLiB library was carried out by Illumina MiSeq with TruSeq-based amplicon preparation. The Library amplicons were prepared by carrying out a PCR with oligonucleotides MiSeq_F & MiSeq_R (Supplementary Table S1) with 2 million beads divided over three 100 μl reactions, consisting each of 1× HF buffer (NEB), 50 pmol of each primer, 20 nmol dNTPs and 2 units Phusion High-Fidelity DNA polymerase (NEB). The PCR reactions were pooled, loaded onto an agarose gel (1.5%), gel extracted and purified by silica columns (Zymoclean Gel DNA Recovery, Zymo Research, Irvine, CA, USA). The amplicon was further processed by the University of Cambridge Department of Biochemistry's Sequencing Facility using the TruSeq kit (Illumina), spiked with 20% PhiX DNA and subjected to MiSeq sequencing (150 base reads, single end).

### NGS analysis

An overview of the analysis approach is provided in Supplementary Figure S7 and accompanying Supplementary Text 1. All software, except IGV (run on Windows 10) and Enrich2 (run as a GUI within Linux Ubuntu), was run from the command line of Linux Ubuntu running within a virtual computer (Oracle VM Virtual Box). Contaminating PhiX sequences and low-quality sequences were filtered from the fastq file using FaQCs version 2.08 (33). Indels and off-target substitution frequency and location was assessed by aligning a reference sequence (corresponding to the sequenced fragment of Z_IgE_) to all reads using a Burrows Wheeler Aligner, BWA-MEM version 0.7.17 (34), with the output SAM file converted to a BAM file, sorted and indexed using SAMtools version 1.7 (35). The location and frequency of off-target substitution and InDels were then determined by IGV version 2.4.14 (36). To prepare for the analysis of codon frequencies in non-InDel containing reads, reads not aligning to the full length of the reference sequence were filtered out by using an AWK command on the BAM file. The filtered BAM file was converted back to fastq format using Bedtools version 2.26.0 (37). To obtain counts of targeted mutations, we used Enrich2 version 1.2.0 (38), while final statistics were prepared with the help of Microsoft Excel. Further details, including command lines, are included in Supplementary Text 1.

### Coupling of Z_IgE_-SpyCatcher to SpyTag-functionalised beads by cell-free expression in emulsion or by using purified Z_IgE_-SpyCatcher protein

Polydisperse water-in-oil emulsions of SpyTag-functionalised, DNA-decorated beads and IVTT were made by pipetting the oil and aqueous phases repeatedly through a 20 μm filter device, until the emulsion appeared homogenous. The filter device was constructed by extracting the filter membrane from a CellTrics cell filtering unit (Sysmex-Partec GmbH, Görlitz, Germany) and fixing this filter between two segments of a 200 μl pipette tip (TipOne, STARLAB UK, Milton Keynes, England), as previously described (25,39). The IVTT-containing aqueous phase (PURExpress, NEB; 12.5 μl, consisting of 5 μl solution A, 3.75 μl solution B, 0.5 μl RNase inhibitor murine (NEB) and 3.25 μl water) and 1–2 million microbeads, were mixed with 8 volumes of oil phase, consisting of a solution of 1% (w/v) fluorinated surfactant RAN (RAN Biotechnologies, Beverly, MA) in HFE7500 oil (3M). The emulsion IVTT was incubated for 1 h at 37°C. To break the emulsion and recover the beads, excess oil phase was removed from the bottom of the tube using a gel saver tip, 100 μl of PBST was pipetted on top of the emulsion, followed by 18 μl of perfluorooctanol (PFO, Alfa Aesar, Heysham, England). This was followed by mixing through vigorous pipetting before the top aqueous layer was transferred to a clean tube on a magnetic rack. To obtain any beads remaining in emulsion, the entire procedure was repeated and the second aqueous fraction was pooled with the first in the tube on the magnet rack. To couple purified Z_IgE_-SpyCatcher protein to SpyTag-functionalised beads, 10 μM of SpyCatcher fusion protein was mixed with 100 000 beads in a total volume of 100 μl for 1 h at 22°C, in PBS. After incubation, beads were washed three times with PBST.

### Binding of IgE-Cy5 to Z_IgE_-SpyCatcher fusion proteins on bead

IgE (native human monoclonal, as provided by Abcam, ab65866) was labelled with Cy5 dye using the Lightning-Link Rapid Kit (Innova Biosciences, Cambridge, UK). As the IgE-Cy5 conjugate was found to be of limited stability at 4°C, care was taken to freeze aliquots of IgE-Cy5 at −80°C, immediately after preparation. IgE-Cy5 labeling of beads was performed in PBST and 30 mg/ml dried skimmed milk powder (Marvel, Premier International Foods, Spalding, Lincs, UK) for 1 h. Beads were washed once with PBST immediately preceding analysis by flow cytometry.

### Flow cytometry-based bead screening & sorting

Flow cytometric analysis was carried out on a FACSSCAN Cytek machine, while flow cytometric sorting of beads was performed on a BD FACSAria Fusion, with four-way sorting into different tubes according to Cy5 fluorescence intensity. The forward and side scatter profile of the beads was used to ensure that sorting was restricted to single beads. Lasers and emission filters for both machines are summarised in Supplementary Table S6. The full-length DNA used to couple to beads to carry out the two separate Affibody screening control experiments presented in Supplementary Figure S9, Figure S10A, B and Figure 5C, is listed in Supplementary Table S4.

### Bio-layer interferometry measurements

Streptavidin Octet tips were equilibrated in PBST with 0.1% BSA. The tips were then loaded with ligand by dipping into a solution of 2.5 μg/ml Avi-tag-Z_IgE_-SpyCatcher for 60 s. The tips were then dipped into a solution of PBST with milk, prepared by mixing skimmed milk powder (to 30 mg/ml, Marvel) in PBST, followed by centrifugation to remove insoluble matter, before the tips were moved into the IgE-analyte containing PBST/milk solution for a 300 s association phase. A dissociation phase (400 s) was subsequently recorded by moving the tips back into the PBS/milk-only solution. To take signal drift into account, data was processed by subtracting the signal from tips which had been loaded with ligand but were not exposed to any IgE during the association phase (one for each Avi-tag-Z_IgE_-SpyCatcher variant, always on the same ‘Octet’ of tips). The Y-axis zero-point was aligned to the start of the association phase. Data were fit to a 1:1 binding model in the Octet Data Analysis Software, assuming only partial dissociation (as we observed in all cases less than complete dissociation, even with the very weak-binding control Avi-tag-Z_IgE_^nonbinder-2^-SpyCatcher).

## RESULTS

### Design and optimisation of SpliMLiB for solid-phase cloning of site saturation libraries

#### Strategy overview

**Figure 1.**
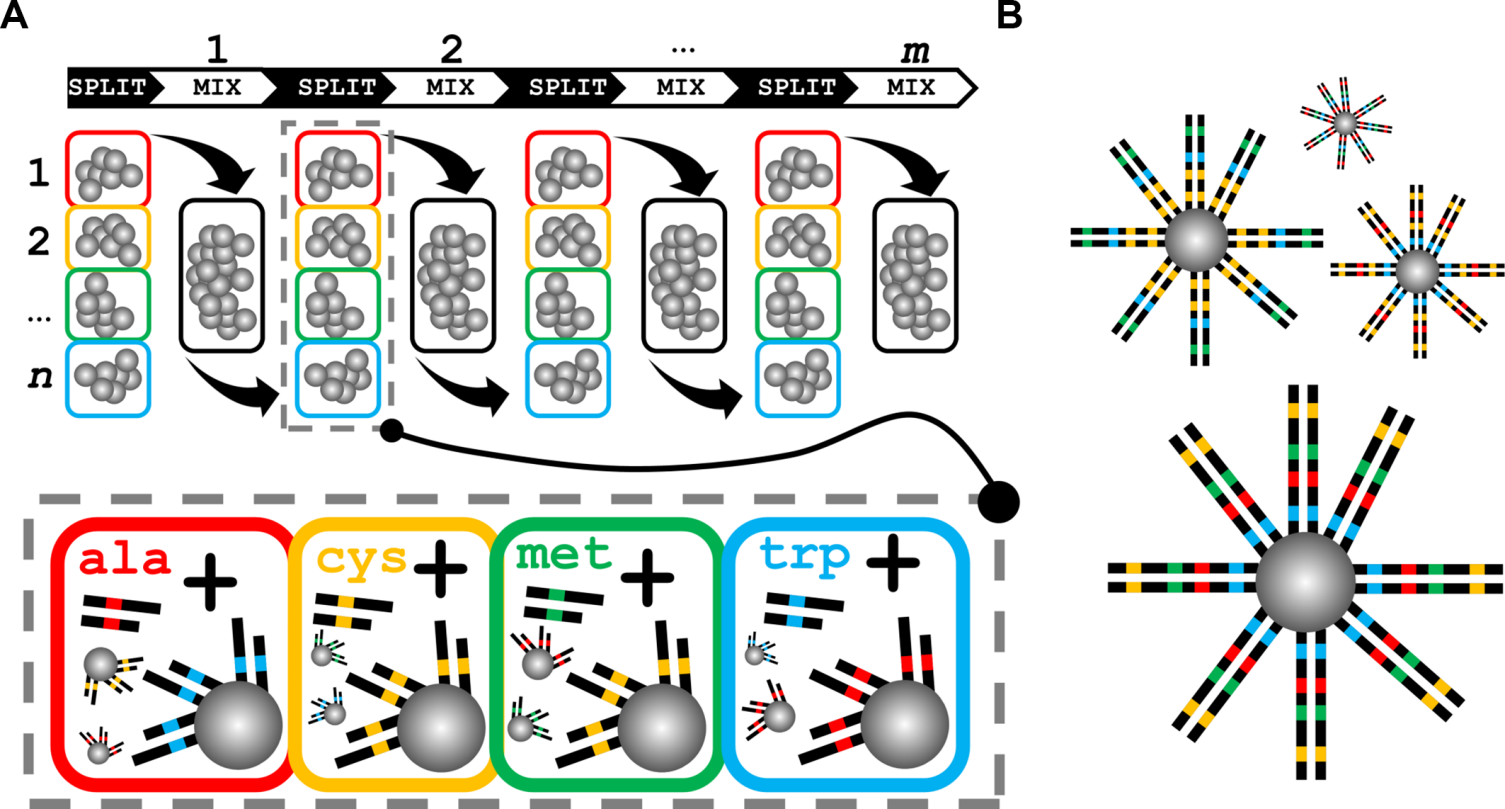
Design of SpliMLiB for solid-phase cloning of site saturation libraries. (**A**) SpliMLiB consists of a number of DNA attachment-rounds, where DNA is immobilised to the bead surface (first attachment-round) or immobilised DNA is extended by ligation (subsequent attachment-rounds). Beads are split into different tubes, with the number of vessels corresponding to the desired number of different amino acid variants at a position of interest within the encoded protein. Beads are mixed between DNA additions, ensuring all combinations of positional variants are achieved. This process may be continued for several attachment-rounds, resulting in a final diversity of *n^m^* where *n* is the number of splits per attachment-round and *m* is the number of attachment-rounds. Each tube within a split receives a DNA fragment carrying a single codon variant, as indicated by the lower dash-lined box shown for the second SpliMLiB round only. (**B**) SpliMLiB results in a site saturation library represented by beads each densely coated in identical DNA.

Our aim was to create a non-degenerate site saturation library, where DNA was densely coated on paramagnetic microbeads, both as a stand-alone method for library generation and as part of microemulsion-enabled bead display of protein variants. Our use of split-and-mix solid-phase cloning achieved both combinatorial diversity and ensured all DNA on any one bead was identical, allowing direct screening of library-encoded protein function. SpliMLiB entails the immobilisation of DNA fragments to split portions of beads, followed by mixing of all beads and addition of a next set of fragments in the subsequent split, extending the bead surface-bound DNA (Figure 1A). Each of the DNA fragments carried a pre-determined, single-variant codon and was added in isolation in each split to a subset of beads. SpliMLiB resulted in a site saturation library represented by beads each densely coated in identical, i.e. ‘monoclonal’, DNA (Figure 1B). Through iteration of the process, a DNA library was generated, the diversity of which corresponded to *n^m^*, where *n* is the number of splits per attachment-round and *m* is the number of SpliMLiB attachment-rounds.

**Figure 2.**
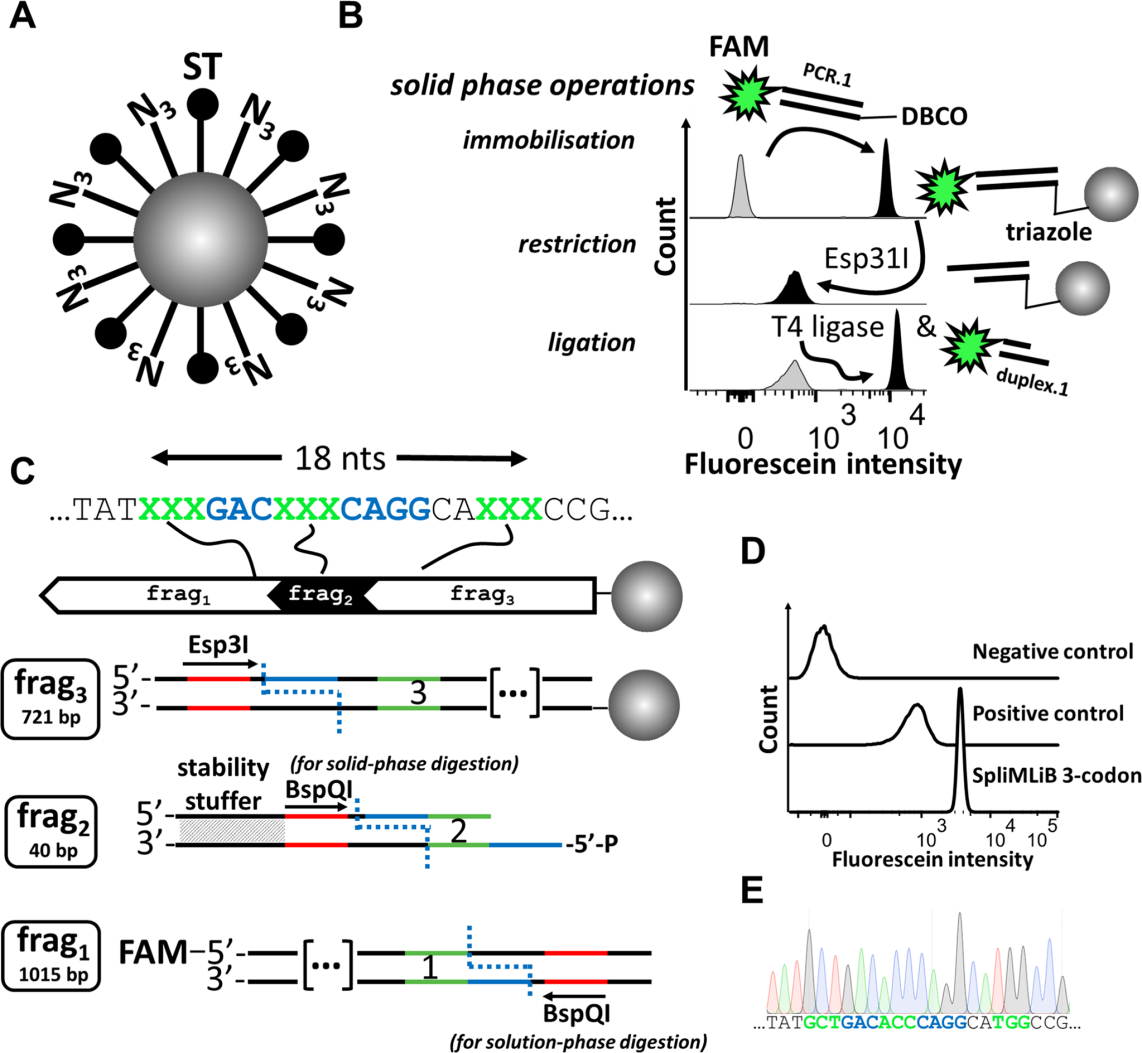
Design of bead surface and solid-phase manipulations of DNA. (**A**) Beads were designed to display both azide (labelled ‘N_3_’) and SpyTag (labelled ‘ST’) moieties (surface modification described in Supplementary Figure S1). (**B**) Flow cytometric analysis of beads for fluorescein-derived fluorescence intensity before (grey) and after (black) immobilisation of fluorescein and DBCO-functionalised DNA (top histogram), after Esp3I treatment (2 hours at 37°C) of the DNA-coated beads (middle histogram) and after exposure of Esp3I-treated beads to a fluorescein-labelled DNA duplex that had a 5′-overhang complementary to the 5′-overhang of bead-immobilised DNA, in T4 DNA ligase buffer, with (black) or without (grey) T4 DNA ligase (bottom histogram). Details of the DNA sequences used for the generation of this panel are set out in Supplementary Figure S4. (**C**) Schematic overview of on-bead assembly allowing potential saturation of three codons in close proximity. The final, bead-attached DNA assembly is shown at the top of the panel, with the three DNA fragments used in the construction are shown below. Restriction sites are depicted in red, target codons in green and sequences used for hybridisation during ligation in blue. The first, PCR-generated amplicon (frag_3_) was attached to bead (*via* copper-free click chemistry) and digested by Esp3I. DNA on the bead was extended using an oligonucleotide duplex (frag_2_) carrying a 5′-phosphorylated cohesive end; the sequence used to ensure stability of the duplex (stability stuffer) prior to ligation is indicated in a diagonal pattern. Once this duplex had been appended to the bead by ligation, a new cohesive end was generated (and stability stuffer removed) through BspQI digestion. Finally, another PCR amplicon (frag_1_), separately prepared with a cohesive end (using BspQI) was ligated to the bead-immobilised DNA. Details of the DNA sequences used for the generation of this panel are set out in Supplementary Figure S5. (**D**) Flow cytometric analysis of untreated beads (top trace), beads carrying full length starting template (i.e. with FAM at one end and DBCO at the other, middle trace) and beads having gone through the 3-codon SpliMLiB process described in C. (**E**) Sanger sequencing chromatogram (templated by a PCR amplicon obtained directly from beads) of the exemplary bead-surface assembled construct shown in panel C where codons to be mutated were designed to be in close proximity (bottom). As in panel C, the green coloring refers to mutated positions, while the blue coloring refers to sequences used for ligations.

To implement this strategy a number of practical challenges had to be met that are addressed in the following paragraphs:

- *Preparation of bead surface for stable DNA and protein attachments*. DNA immobilisation on beads was required to be of sufficient stability to guarantee the integrity of the library from its build-up, through to microemulsion-enabled bead display screening and recovery of hits. Conventionally, biotinylated DNA is immobilised onto streptavidin-coated beads, although the limited stability of the biotin–streptavidin complex after chemical conjugation to streptavidin and in different solvent conditions is increasingly recognised (40). We discovered that the linkage between biotinylated DNA and streptavidin beads was perturbed by the *in vitro* expression mixture used in our bead screening stage (Supplementary Figure S3A), consistent with an earlier observation of weakened interaction between biotinylated DNA and streptavidin beads in transcription experiments (41). Therefore, we designed a custom surface coating of the beads with azide, allowing covalent coupling of DNA to beads using strain-promoted copper-free click chemistry (42). We also included SpyTag on the bead surface, to support the attachment of protein variants (fused to SpyCatcher) via isopeptide bond formation (43), during later droplet screening of SpliMLiB. To produce azide and SpyTag-functionalised beads (Figure 2A), a soluble, tetrameric carrier protein (Tamavidin-2-HOT (44)), fused to SpyTag peptide, was chemically crosslinked to the bead surface (Supplementary Figure S1A). Remaining free lysine side chains of bead-immobilised carrier protein were functionalised with azido-PEG4-NHS (Supplementary Figure S1B), allowing dibenzocyclooctyne (DBCO)-functionalised DNA immobilisation through a covalent linkage (a triazole) between the DNA and the beads (Supplementary Figure S1C).The bead-surface immobilisation of a dsDNA fragment that was functionalised with DBCO at one 5′-end and fluorescein at the other 5′-end was found to occur with high efficiency and specificity, as monitored by flow cytometry (Supplementary Figure S1D & Figure 2B, top histogram). Importantly, while biotinylated DNA was rapidly lost from beads upon exposure to IVTT (92% loss after 1 hour in IVTT), DBCO-functionalised DNA displayed increased stability (41% loss after 1 hour in IVTT) (Supplementary Figure S3). At the protein level, immobilisation of GFP-SpyCatcher proceeded with excellent efficiency and specificity (Supplementary Figure S1E).
- *Cohesive end generation*. Next to stable DNA immobilisation, a second factor affecting the final yield of SpliMLiB was the efficiency of restriction. To avoid introducing any undesired sequence into the final library, we used Type IIs restriction enzymes Esp3I and BspQI, allowing scar-free cloning as the digestion takes place outside of the enzyme's recognition site. We initially faced the ‘suicidal’ terminal end problem: DNA fragments that extend a growing chain on the solid surface irreversibly end further extension if they do not carry a 5′-overhang at their far end. This problem, previously described by others (45), was solved with a simple tweak to the protocol: treatment of the entire bead pool with restriction enzyme, rather than digesting DNA off-bead. Treatment with Esp3I was found to lead to digestion of 94% of DNA, as monitored by loss of bead-immobilised DNA terminally labelled with fluorescein (Figure 2B, middle histogram).
- *Ligation efficiency*. An important factor for SpliMLiB yield was the efficiency of the solid-phase ligation step. During an early phase in the optimisation of the SpliMLiB protocol, we faced low yields of solid-phase ligation (not shown) and erroneously ascribed that to poor ligation efficiency. However, we soon realised that this situation was a consequence of poor efficiency of the upstream step in the protocol, the solid-phase restriction (see above). Fortunately, we found solid-phase ligation not to require any optimisation, as long as i) DNA carried appropriate overhangs (assured through solid-phase digestion) and ii) sufficient solution-phase DNA was provided (20 million DNA molecules per bead). Ligation efficiency was monitored using a fluorescein-labelled dsDNA with a 5′-overhang complementary to the 5′-overhang of DNA immobilised on the beads. In the presence of T4 DNA ligase, such beads displayed the same fluorescence intensity as beads to which fluorescein-labelled DNA had been attached directly via click chemistry (Figure 2B, bottom histogram), suggesting efficient ligation. Unspecific binding of DNA to beads during the ligation reaction was excluded by the observation of a lack of increase in fluorescence in beads receiving fluorescein-labelled DNA with correct complementary 5′-overhang, but no T4 DNA ligase (Figure 2B, bottom histogram).
- *Saturation of proximal codons*. Finally, we assessed SpliMLiB's suitability for the saturation of codons in close proximity (i.e. separated by fewer bases than found in even a short, 20-mer oligonucleotide) in a non-degenerate manner, where mutant codons are carried on separate input fragments. Combining for instance three codons on the same input fragment, generated by conventional, phosphoramidite monomer-based synthesis and in a non-degenerate manner, would have required the use of 8000 oligonucleotides, an impractical prospect. We introduced a key design feature, the incorporation of a terminal ‘stability stuffer’ element in the incoming DNA duplex, to ensure the stability of the incoming DNA duplex and thus its acceptance by T4 DNA ligase (46). Type IIs recognition sites allowed scarless removal of the stuffer and introduction of a ssDNA overhang for the next SpliMLiB attachment-round. To test this approach, we designed a scheme for the potential saturation (only a single split per fragment was carried out) of three closely situated codons, where DNA was split into two longer, flanking, PCR-generated fragments (Frag_1_ & Frag_3_) and a central fragment prepared by duplex formation of two oligonucleotides (Frag_2_, Figure 2C and Supplementary Figure S5). The central fragment was 40 bp in length but contributed just 3 bp (i.e the targeted codon) and 7 bases (the 4 & 3 nt-ssDNA cohesive ends). Using this strategy, the DNA assembled with high efficiency (Figure 2D) and DNA directly amplified and sequenced from the 3-codon SpliMLiB beads showed a perfect Sanger chromatogram (Figure 2E). Thus, SpliMLiB permits the targeting of codons separated by only a single, intervening, constant codon.

Taken together, our approach thus permits the assembly of DNA on beads, where the DNA-surface attachment is highly stable, where each addition of DNA fragment proceeds with excellent efficiency and where codons in close proximity can be individually targeted. By exploiting the combinatorial diversification arising out of a split & mix approach during the rounds of DNA appendage, DNA libraries can be constructed, which benefit from being represented by ‘clonal beads’.

### Construction of a 160 000-membered library for Z_IgE_ affinity maturation and validation of library diversity by NGS

#### Library assembly

**Figure 3.**
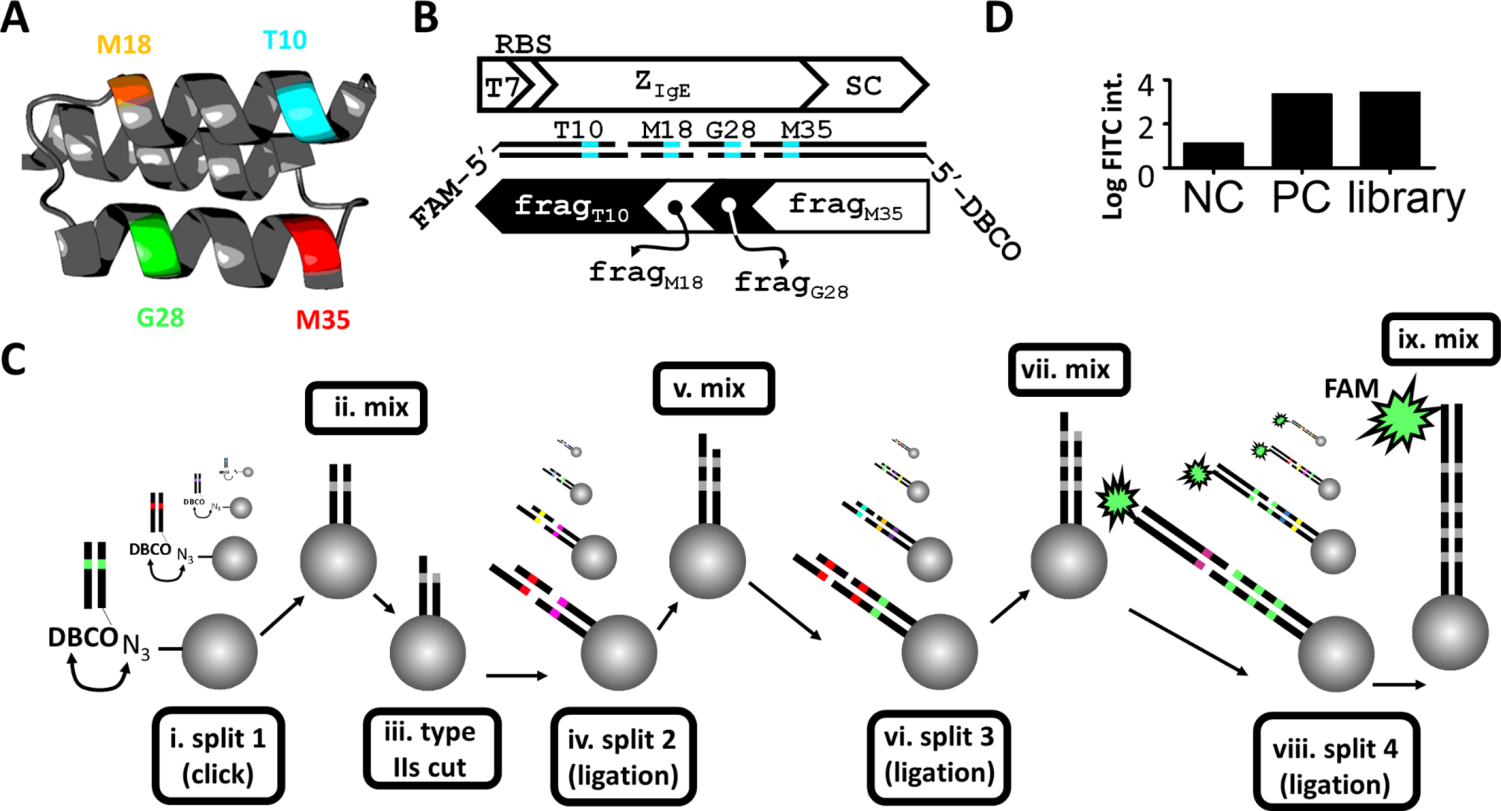
Design and workflow of a SpliMLiB library for Z_IgE_. (**A**) Model structure for Z_IgE_ (modelled by Swissmodel (118), based on a template with PDB ID 2m5a (119), indicating the locations of the four positions targeted in the SpliMLiB library. (**B**) Schematic overview of the final Z_IgE_ expression construct that was assembled in four SpliMLiB attachment-rounds. The Z_IgE_ sequence was divided into four sets of fragments, each of which carried one of the targeted positions. These SpliMLiB input fragments were generated either by PCR (fragment sets frag_T10_ & frag_M35_) or through annealing of partially complementary oligonucleotides (fragment sets frag_M18_ & frag_G28_). The first set of fragments to be immobilised, frag_M35_, was functionalised with DBCO, allowing immobilisation of fragments through copper-free click chemistry to azide-functionalised beads. The last set of fragments to be ligated, frag_T10_, was functionalised with FAM, allowing monitoring of the efficiency of total SpliMLiB library assembly efficiency. The Esp3I type IIs sites included on the ends of the PCR-generated fragments supported seamless ligations to the oligonucleotide duplexes which had 5′-overhangs by design and which had been enzymatically 5′-phosphorylated. (**C**) The SpliMLiB workflow is schematically depicted. In a first attachment-round, DNA was immobilised on split populations of beads using copper-free click chemistry (i), before beads were mixed (ii) and subjected to an on-bead restriction reaction (iii) in order to generate a 5′-overhang. Next, beads were split again and 5′-phosphorylated synthetic duplex DNA with a 5′-overhang complementary to the 5′-overhang (generated in step iii) was ligated to the bead-immobilised DNA. After subsequent mixing (v) and splitting of the beads, the bead-bound DNA was ready for extension by yet another 5′-phosphorylated synthetic duplex DNA fragment (vi). Beads were then mixed (vii) and split for the final ligation (viii) to add a PCR fragment carrying a 5′-overhang (generated by off-bead type IIs restriction), complementary to the penultimate fragment, the 5′-phosphorylated synthetic duplex DNA. Each PCR amplicon from this last set of fragments was labelled with a 5′-FAM at the far end, for flow cytometric analysis of the mixed final library (ix). (**D**) The efficiency of SpliMLiB library construction was analysed by flow cytometry. The positive control (PC) was prepared by immobilising the full length Z_IgE_ DNA fragment by click chemistry on the beads (identically end-labelled with fluorescein as the library bead DNA). Untreated beads that did not contain any DNA served as the negative control (NC).

The utility of the SpliMLiB system was probed by building a library for the affinity maturation of an Affibody protein binder recognising the IgE antibody. Affibody molecules are small, three-helix bundle antibody mimetics with improved stability and expression compared to classical antibodies (47). Affibody Z_IgE_ had been selected by phage display, based on a degenerate codon (VNN) library targeting 13 different positions, with a reported *K*_d_ of 0.4 μM (48). We reasoned that the original phage display library must have undersampled the theoretical amino acid sequence space implied by the randomisation scheme (16^13^), encouraging us to seek to improve the affinity of this binder by a more targeted and balanced mutagenesis library using SpliMLiB. Out of the 13 sites originally randomised, four were chosen as SpliMLiB targets: T10, M18, G28 and M35 (Figure 3A). Each of these sites were to be fully saturated, resulting in a theoretical diversity of 20^4^, i.e. a 160 000-membered SpliMLiB library. The design of the library entailed four different DNA fragments, each of which was generated in sets of twenty different variations, for each of 20 different codons at the targeted sites. A first set of DBCO-modified fragments (frag_M35_, for direct immobilisation to the bead surface) was generated by PCR, varying at the M35 position and encoding a C-terminal SpyCatcher sequence, to support later covalent linkage of expressed protein variants to the SpyTag-functionalised beads. The two sets of central fragments (frag_G28_ and frag_M18_) were generated through hybridisation of partially complementary oligonucleotides, varying at the G28 and M18 positions respectively. The fourth and final set of fragments (frag_T10_) was generated via PCR, varying at the T10 position (Figure 3B and Supplementary Figure S6), while also introducing a T7 promoter and ribosome binding site (RBS) for later *in vitro* expression (see below). Library build-up was conducted in the antisense direction. Thus, any incompletely extended fragments would not contain the T7 promoter or ribosomal binding site, mitigating the risk of impairment of transcription and translation efficiency of full-length DNA during subsequent cell-free expression. The full workflow entailed the design of oligonucleotides, the preparation of PCR fragments and oligonucleotide duplexes and the split & mix-based processing of beads (Figure 3C). SpliMLiB library synthesis was started with 20 million beads, of which 8.2 million remained for NGS analysis, expression and screening after the final ligation step (accounted for by inevitable bead loss during washing steps). The efficiency of DNA library assembly on the beads was confirmed using flow cytometry by comparing the fluorescence signal obtained from beads coated with a fluorescein-labelled, full-length DNA fragment to the pooled library beads after ligating the final, fluorescein-labelled fragment (Figure 3D).

#### Deep sequencing of library

**Figure 4.**
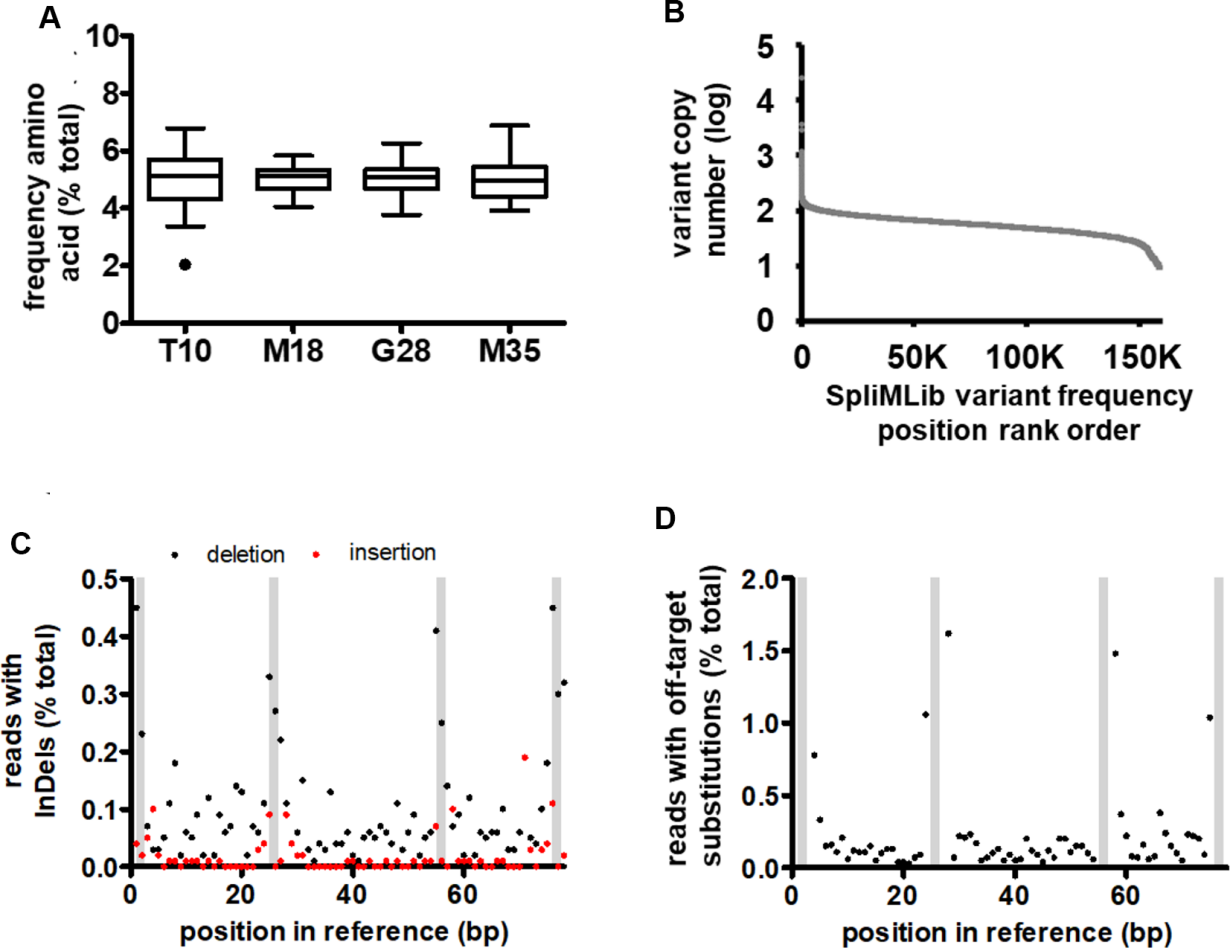
Analysis of Z_IgE_ SpliMLiB library by NGS. (**A**) Box and whiskers plots for the frequency of all 20 amino acids at each of the four target sites. As per convention, the Tukey whiskers are extended along 1.5 times the interquartile distance or to the highest/lowest point, whichever is shorter. The sole data point outside the range of the whiskers (for T10P) is indicated by a black dot. (**B**) Frequency distribution of all theoretical library variants arranged in order of frequency with which they were observed in NGS. (**C**) Frequency of insertions and deletions occurring at each position of the sequenced fragment from the SpliMLiB library. (**D**) Frequency of off-target substitutions occurring at each position of the sequenced fragment from the SpliMLiB library. In panels C & D, shaded bars represent the positions of the four targeted codons (from left to right, T10, M18, G28 and M35).

To validate the quality of the library generated using the SpliMLiB technique, the Z_IgE_ input library was sequenced on the Illumina MiSeq platform. A PCR fragment was produced from the input beads covering all four targeted sites on the amplicon for 150 base single-read sequencing, resulting in a sequencing depth of 89 times the theoretical library size (14.2 million reads, Supplementary Table S7). We first analysed those reads not containing InDels (86.2% of all reads). We found that the distribution of individual amino acids at each of the four positions indicated a balanced distribution, with a per amino acid frequency over all four targeted positions of 5.1% ± 0.77 (median ± standard deviation) (Figure 4A and Supplementary Table S9). Similarly, there was excellent coverage of the total theoretical library size, with 99.3% of the theoretical library members encountered in the NGS data (Figure 4B). Although there were two small subsets of theoretical variants that were either over-represented or under-represented, 88% of all observed variants were found to vary by <2-fold in copy number from the average read number and 96% varied by <3-fold from that same value. Further quality control of the library was undertaken by analysing for the presence of off-target substitutions, deletions, insertions and truncations. We identified 2.0 million reads (13.8% of the total reads) that had insertions, deletions and/or truncations (Table S8). Of these indels, the majority concerned deletions (Figure 4C) and truncations (Supplementary Figure S8). Interestingly, InDels appeared to be more prevalent close to the sites targeted for saturation. Similarly, off-target substitutions occurred more frequently close to targeted sites (Supplementary Table S8 and Figure 4D). There was a small but significant contamination by wild-type sequence, amounting to 0.25% of the sample. This sequence likely represented carry-through from the wild-type template used in PCR reactions to generate frag_T10_ and frag_M35_. In summary, sequence analysis strongly suggested the SpliMLiB Z_IgE_ library was near-complete and unbiased.

### Instant protein screening platform

#### Cell-free protein binder screening

**Figure 5.**
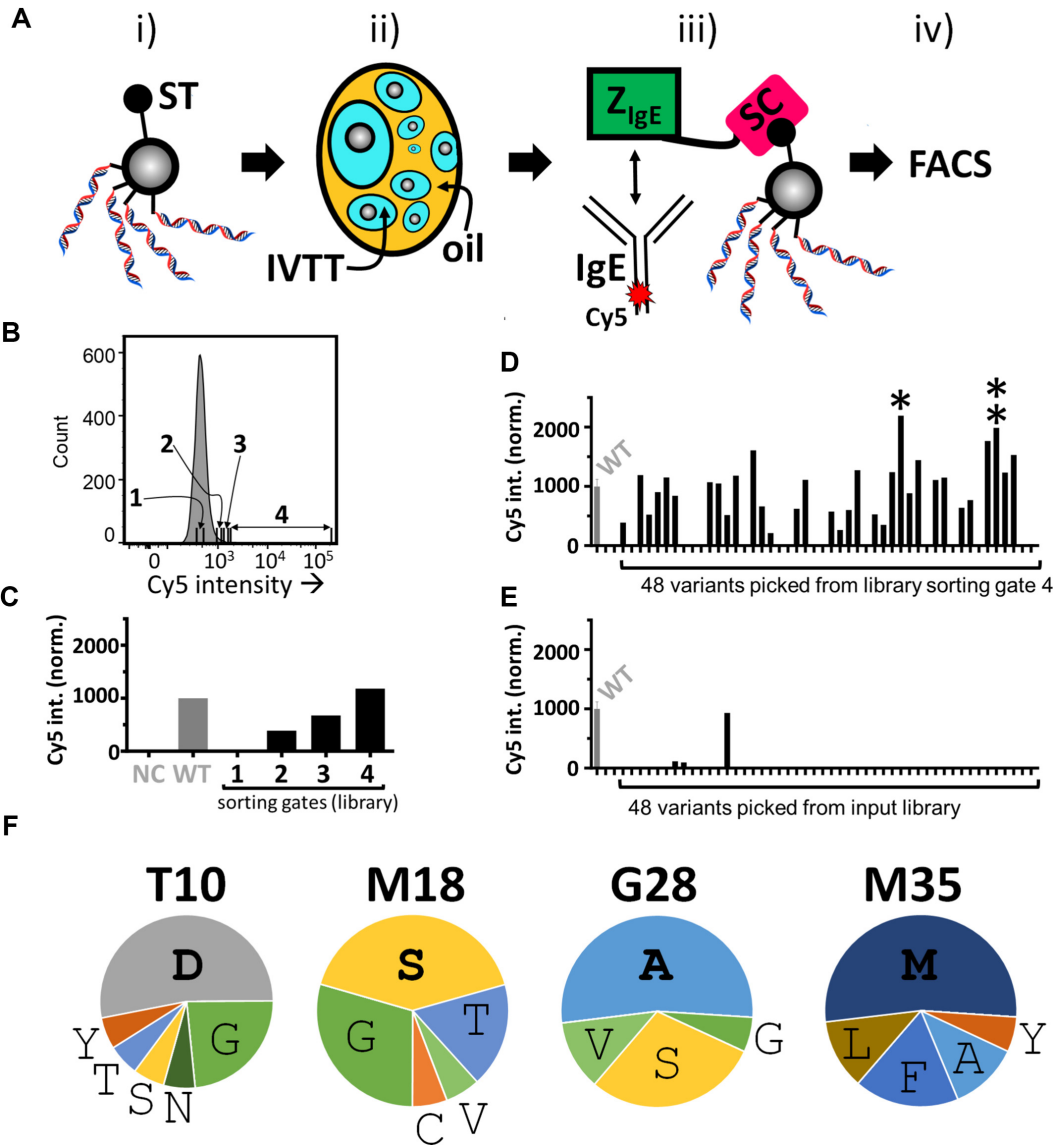
Microemulsion-based bead display screening of the Z_IgE_ SpliMLiB library. (**A**) Schematic overview of a round of SpliMLiB-enabled directed evolution of Z_IgE_. SpliMLiB beads (i) were singly encapsulated in emulsion IVTT at 37°C for 1 h (ii), sufficient time to allow for both Z_IgE_-SpyCatcher variants’ expression as well as for their SpyTag-SpyCatcher-mediated immobilisation on the bead surface, after which the emulsion was broken, and the washed beads were exposed to Cy5-labelled IgE (iii), followed by flow cytometric sorting of beads based on Cy5 signal (iv). (**B**) Representative histogram recorded during the flow cytometric sorting of SpliMLiB Z_IgE_ library beads. The range of fluorescence intensity used for each of the sorting gates 1–4 is indicated. (**C**) Analysis of pooled, recovered and subcloned DNA from the sorting gates set out in panel B. DNA was used to express protein in IVTT under bulk, i.e. non-emulsion conditions, in the presence of SpyTag-functionalised microbeads. The microbeads, having captured the SpyCatcher fusion proteins, were then incubated with 200 nM IgE-Cy5 and analysed by flow cytometry. Cy5 fluorescence intensity was normalised to a sample prepared from beads exposed to purified Z_IgE_^wild-type^-SpyCatcher protein (WT, grey bar). Negative control (NC) was beads not exposed to any Z_IgE_-SpyCatcher protein. (**D**) Analysis of bacterially expressed & purified variants derived from the stringently sorted library output from FACS sorting gate 4. Beads that had been bound with Z_IgE_-SpyCatcher variants were incubated with 200 nM IgE-Cy5 and analysed by flow cytometry. Z_IgE_^wild-type^-SpyCatcher (labelled WT) was included as control and was used to normalise all fluorescent values. The variant showing the highest Cy5 median signal (variant 33, marked by a single asterisk) and second highest (variant 44, marked by a double asterisk) signal were taken forward for further analysis. (**E**) As panel D, except for 48 randomly picked clones derived from the unsorted SpliMLiB input library beads. (**F**) Frequencies of amino acids encountered in selected variants displaying a higher binding signal than Z_IgE_^wild-type^-SpyCatcher (17 in total). The most frequent amino acid at each position is indicated in bold to emphasise it.

A unique and powerful feature of SpliMLiB is the generation of *monoclonal* beads, each carrying many copies of a single library variant. This feature allows direct expression and screening of the encoded proteins, generating one-bead-one-protein libraries. To put this into practice, a scheme was devised to screen Z_IgE_ protein variants using the SpliMLiB library described above. SpliMLiB beads were encapsulated in the droplets of a water-in-oil emulsion, with *in vitro* expression mix in the aqueous phase. As Z_IgE_ was fused to SpyCatcher, the expressed protein variants became covalently attached to the SpyTag-functionalised SpliMLiB beads, *via* an isopeptide bond (49), leading to a highly stable genotype-phenotype linkage. Thus, upon de-emulsification of the beads and incubation with Cy5-labelled IgE, the genotypes of the sorted Z_IgE_ molecules could be sequenced (Figure 5A). To ascertain that the Z_IgE_ SpliMLiB library format could be integrated with screening experiments, we carried out control experiments (Supplementary Text 2), to confirm the stability and lack of cross-contamination of the emulsion IVTT (Supplementary Figure S9) and the successful enrichment of functional binders (Supplementary Figure S10).

#### Screening of SpliMLiB Z_IgE_ library

Having validated the instant bead screening protein selection platform, we screened the fully randomised, 160 000-member SpliMLiB Z_IgE_ library. Four million SpliMLiB Z_IgE_ beads were subjected to emulsion IVTT, exposed to 200 nM of IgE-Cy5 and FACS sorted into four different gates of increasing stringency (Figure 5B). DNA was recovered by PCR and cloned into an acceptor vector. Promisingly, pooled analysis of the sorting gates showed that increasing sorting stringency correlated with an increasing IgE-Cy5 signal (Figure 5C). To characterise individual hits, a subset of single clones was picked from the most stringent gate (containing ∼800 beads), expressed in small scale *E. coli* BL21(DE3) cultures and purified with Ni^2+^-affinity chromatography. Most (45 out of 48) clones resulted in ample soluble and pure protein (∼0.15–1 mg protein from 20 ml culture), as analysed by SDS-PAGE (Supplementary Figure S11A). These protein variants were loaded on beads (*via* SpyTag-SpyCatcher bonding) and analysed for binding to Cy5-labelled IgE (Figure 5D and Supplementary Table S10). We found that despite the presence of 15 (31%) false-positive, non-functional clones (i.e. displaying <20% of the wild-type binding signal), 16 (33%) clones were found to be functional (i.e. displaying >20% of the wild-type binding signal) and 17 (35%) variants (including hits 33 and 44, marked) appeared to be better binders than the wild-type (Figure 5D). When the same number of clones from the unsorted library was analysed, a strikingly different picture was revealed: 47 (98%) of clones were found to be non-functional, a single (2%) clone was found to be functional and no clones improved over wild-type were revealed (Figure 5E and Supplementary Table S11). Thus, a single round of bead display selection of the SpliMLiB Z_IgE_ beads was sufficient to enrich for functional and affinity-improved hits. To discern patterns of enrichment within the binding subset of selected protein variants, Sanger sequencing was carried out on all 48 characterised clones (Supplementary Table S10), but we focused our analysis on the 17 variants that displayed a higher binding signal than wild-type (Figure 5F). At each position, one residue was encountered most frequently (9, 7, 9 and 7 times at positions T10, M18, G28 and M35, respectively, Supplementary Table S10). At position T10, aspartic acid was mainly found, while at position M18 serine was favoured, with similarly small residues glycine and threonine were also allowed. The G28 position was dominated by alanine, with serine as the second most commonly found residue. Finally, at position M35, methionine (i.e. the wild-type residue) represented the predominant amino acid encountered, with several more relatively hydrophobic residues also encountered.

#### A tighter binding consensus mutant

The most commonly occurring mutation at each of the four positions was T10D, M18S, G28A and M35M, respectively (Figure 5F). Interestingly, we did not encounter the combination of all four of these mutations in any of the 48 characterised variants, although inspection of the input NGS sequences confirmed that the consensus mutant had also been available in the original library. To investigate whether this consensus combination might represent a tighter binding variant, Z_IgE_^consensus^-SpyCatcher was constructed using SpliMLiB DNA fragments frag_T10D_, frag_M18S_, frag_G28A_ and frag_M35M_ and the same solid-phase DNA assembly method used in the construction of the Z_IgE_ library. Biolayer interferometry (BLI) measurements of purified proteins (Supplementary Figure S12) confirmed that Z_IgE_^consensus^-SpyCatcher was improved over Z_IgE_^wild-type^-SpyCatcher, as well as over the two top hits from the bead display selection: Z_IgE_^33^-SpyCatcher and Z_IgE_^44^-SpyCatcher (Table 1).

**Table 1.** Affinity characterisation of selected Z_IgE_-SpyCatcher variants by biolayer interferometry (BLI). To prepare for biolayer interferometry (BLI) measurements, these four variants (in addition to a variant with alanine mutations at all four SpliMLiB-targeted sites, Z_IgE_^nonbinder-2^-SpyCatcher) were sub-cloned to an expression vector allowing site-specific biotinylation of a lysine on the short N-terminally fused BirA tag (Supplementary Figure S2E). Binding constants were estimated by fitting of the obtained BLI data to a 1:1 binding model assuming only partial dissociation (Supplementary Figure S12). Provided are the mean *K*_d_ values from measurements at three different ligand (IgE) concentrations, together with the standard error. ^1^A fit could be obtained only for the highest concentration of IgE, precluding an accurate estimation of Z_IgE_^wild-type^-SpyCatcher affinity. Similar difficulties with Affibody affinity determination using surface plasmon resonance have been noted elsewhere (120)

	Mutations				
Z_IgE_-SpyCatcher variant	10	18	28	35	*K*_d_ (μM)
Z_IgE_^wild-type^	T	M	G	M	∼7.3^1^
Z_IgE_^33^	G	S	A	M	2.1±0.5
Z_IgE_^44^	D	G	S	F	4.8±1.3
Z_IgE_^consensus^	D	S	A	M	0.61±0.06

## DISCUSSION

### Straightforward generation of fully non-degenerate libraries with SpliMLiB

As a technique to generate site saturation libraries, SpliMLiB offers several advantages over existing methods (Table 2). Library quality may be adversely affected by poorly controlled codon frequencies. SpliMLiB provides fully balanced and non-degenerate codons at each position, thereby maximising the chance of success in a screening campaign, by ensuring no part of sequence space is omitted, even small areas of which may encode the desired phenotype. Our NGS analysis of the 160,000-membered Z_IgE_ SpliMLiB library revealed well-balanced codons so that full inclusion of all 20 natural amino acids was reliably achieved. Had the same positions in our Z_IgE_ target protein been saturated using the commonly employed NNK, it would have taken a greater effort to fully screen as there would have been 6.5 times more theoretical variants and the library would have been less balanced in terms of amino acid representation. Thus, although a plethora of highly efficient techniques are available for straight-forward library construction (6–8), such libraries may not offer the most economically sensible route (9), especially when the cost of screening is high.

**Table 2.** Examples of codon diversification approaches, advantages and limitation as wells as specific implementations and embodiments. ^1^These are not intended to be exhaustive and the reader is referred to excellent, comprehensive reviews such as (121)

Codon diversification	Mutagenic effect	Advantages / limitations	Implementation examples^1^
NNK, NNS (7)	32 codons	Simple, cheapest oligonucleotide synthesis /High degeneracy	OmniChange (17)
22C & other small-intelligent approaches (10,11,50)	Semi-non-degenerate	Simple oligonucleotide synthesis/ Unsuitable for proximal codons	Darwin Assembly (7)
TRIM technology (51)	Fully non-degenerate	Proximal codons targetable/ Expensive & custom codon ratios not available	Controlled randomisation (68)
Phosphoramidite synthesis with orthogonal groups (53) or with Resin Splitting (52)	Fully non-degenerate	Control at the nucleotide level over randomisation / laborious, expensive and requiring large amounts of reagents	Custom randomisation ratios at proximal codons (56)
MAX randomisation (59)	Fully non-degenerate	Cheap reagents, protocol and workflow / cannot target more than two proximal codons	Zinc finger screening (60)
Stepwise extension of gene by successive ligations with fully defined mixtures of codons	Fully non-degenerate	Allows targeting of consecutive proximal sites /labour intensive or requires automation	Slonomics (15), ProxiMAX (61) & Colibra (16)
Solid-phase split & mix ligation of DNA duplexes and amplicons	Fully non-degenerate	Directly screenable format / at least one constant codon required between two saturated residues	SpliMLiB (this study)
Microarray & full gene synthesis	Fully non-degenerate/entire homologues	Full control over entire sequence / Currently limited to ∼10^4^ variants	DropSynth (64), mini-proteins (66,67)

The codon bias problem has been partially addressed by the ‘22c-trick’ and other approaches towards small and smart libraries (10,50), where blends of several different oligonucleotides provide near-equal distribution of all amino acids (11). Due to the need for multiple oligonucleotides per position, however, targeted positions must be at least mutagenic oligonucleotide-lengths apart, even though it is often desirable to target multiple, proximally located amino acids, e.g. in reshaping the active site of an enzyme. In contrast, SpliMLiB allows saturation of codons in close proximity of one another, separated by just a single constant codon. The practical solution provided by SpliMLiB is based on use of a Type IIs restriction enzyme that scarlessly cuts away a portion of DNA that initially provides the stability to a DNA duplex, necessary for T4 DNA ligase activity.

Other technologies have been developed that can deliver fully non-degenerate site saturation, even of successive, proximal codons (Table 2). TRIM technology, where defined blocks of trinucleotide phosphoramidites are incorporated during synthesis (12), enables fully non-degenerate site saturation but is expensive due to the additional challenges involved in working with the necessary protecting groups during the synthesis of the trinucleotide itself and during its subsequent use in phosphoramidite synthesis (51). The ‘split-resin’ approach achieves randomisation by carrying out split & mix phosphoramidite synthesis of oligonucleotides. This method is, however, difficult to automate, suffers from poor yield, requires facilities not generally available in most biochemistry laboratories (52,53) and has thus seen only limited applications (54–56). Another strategy implemented at the oligonucleotide synthesis stage, involves the use of orthogonal protecting groups on monomer phosphoramidites, similarly giving full control over codon randomisation (57). In SpliMLiB on the other hand, the entire library may be built up from relatively inexpensive, desalted, chemically unmodified oligonucleotides, except for a single, common, modified oligonucleotide to allow library DNA immobilisation. The end-user prepares SpliMLiB input fragments with routine manipulations such as enzymatic 5′-phosphorylation, oligonucleotide duplex generation or PCR fragment generation. Thus expensive oligonucleotides with base modifications such as uracil (58) or trimer codon mixes are avoided. Like SpliMLiB, MAX mutagenesis, where NNN-containing ‘template’ oligos are hybridised to oligonucleotides containing specific codons complementary to the NNN part, is straightforward to implement and does not require expensive reagents (59,60). However, the MAX technique requires at least two constant codons between every saturated codon (59).

A shared technical feature of the library-generating platforms ProxiMAX (61) and Slonomics (15) is the successive ligation of small portions of the gene. In the ProxiMAX technique, variant codon-introducing oligo duplexes (or hairpins) are blunt-end ligated to a growing template, enabling fully non-degenerate library synthesis (61). The ProxiMAX method requires a PCR workup step between every codon addition, as well as being subject to differences in codon-dependent ligation efficiency, necessitating careful adjustments of variant concentrations (16,61). Although this technology has since been improved (and renamed as ‘Colibra’), it now requires specialist robotic pipetting equipment, limiting its widespread adoption in library-generating laboratories (16). Similarly, the Slonomics approach, while capable of providing high quality libraries through successive nucleotide triplet build-up, is effectively a proprietary, robotics-based technique, requiring a 4096**-**set of ‘splinkers’ as input material (15,62). By contrast, the SpliMLiB methodology is more straightforward to implement in any molecular biology lab, avoiding robotic equipment and requiring nothing more sophisticated than paramagnetic microbeads and a bar magnet. More recently, oligonucleotides synthesised on microarrays have been used to assemble gene libraries, typically by polymerase cycling assembly (63). However, due to the need to synthesise the entire gene length, the price of gene synthesis, which remains stubbornly high for reasonably error-free DNA, means this technique is limited to the synthesis of maximally ∼3 × 10^4^ variants (63), including homologues (64), designed shuffling libraries (65) and short proteins (66,67), while we demonstrate here that SpliMLiB gives access to a library size >10^5^ and is limited by transformation efficiency only (rather than synthesis).

We were reassured by the fact that previous work had established that solid-phase ligations can be very efficient (effectively 100%, as later shown by our flow cytometry experiments), from the addition of very short oligonucleotide duplexes (46), through to the ligation of multiple kilobase fragments of DNA (45,69). Indeed, there is even a commercially exploited gene synthesis technique involving successive solid-phase ligations of oligonucleotide complexes (70). We found that important parameters for optimal DNA solid-phase assembly included the amount of DNA immobilised onto beads (we recommend 10^7^ molecules of DNA per bead), the provision of a stabilised DNA duplex for ligation and carrying out of the restriction enzyme digestion on already immobilised DNA where possible. Furthermore, the occasional use of a fluorophore (via attachment at the 5′-end of an oligonucleotide) at the growing end of the DNA allows quantitative monitoring of assembly success during library build-up. The carefully documented SpliMLiB optimisation experiments will facilitate implementation of the technique in laboratory practice. Furthermore, the NGS revealed the library to provide good coverage of the total theoretical diversity: 99.3% of all theoretical variants were represented. The SpliMLiB library was by no means perfect, as we detected a significant number of errors, including off-target substitutions and deletions (13%). Nevertheless, this error rate was an acceptable price to pay in return for a well-balanced, non-degenerate library with a reasonably straightforward method to generate it.

The limits of SpliMLiB are defined by the maximum number of targeted sites and the number of splits per site in DNA attachment rounds. Given the efficiency with which four fragments were immobilised and ligated in the Z_IgE_ SpliMLiB library, ligation of twice the number of fragments would seem conceivable, leading to library sizes approaching 2.5 × 10^10^. Libraries can also be constructed to have maximal functional diversity by allocating available diversity over different positions (e.g. ∼160,000 variants used either for full site saturation at four sites or by allocating 11 different amino acids at five sites). Although we have found it useful to follow SpliMLiB library synthesis by flow cytometry, especially as doing so helped us to identify several critical factors requiring optimisation (e.g. carrying out restriction on pre-immobilised DNA), it may be more convenient and economical to use amounts of DNA (we typically supplied 5 million molecules of DNA per bead) that are no longer sufficient to be monitored directly by flow cytometry of beads (i.e. less than 10^7^ molecules of DNA per bead) but that could still be followed by real time or end point PCR.

### SpliMLiB compared to ‘DNA-encoded libraries’

‘DNA-encoded libraries’ (DELs) can be considered conceptually similar to the SpliMLiB approach we introduce here: successive additions of building blocks by synthetic chemistry are encoded by parallel additions of known DNA sequence, in combinatorial split & mix fashion, to create diverse collections of small molecules that can be identified by sequencing the attached DNA (31,71). Encoding DNA may be attached to library molecules through a small chemical linker (72). Alternatively, both may be attached to a bead, resulting in a combination of the ‘one-bead, one-compound’ approach (30,73) with the DEL approach, leading to many copies of DNA per bead (and thus per hit), improving the chance that viable, PCR-amplifiable templates remain after the chemical synthesis steps (74). As in SpliMLiB, DNA may be added as oligonucleotide duplexes, using T4 DNA ligase to create stretches of PCR-amplifiable DNA (74). DELs allow screening of compound libraries, generated using building blocks and synthesis schemes inaccessible through ribosomal protein synthesis, for binding to a protein target (75–78). Certain DEL formats can be screened using a water-in-oil emulsion-based compartmentalisation strategy called ‘binder trap enrichment’. A protein target and a small molecule ligand are tagged with DNA barcodes and initially mixed in a free solution. Subsequently, individual, bound complexes are brought into emulsion, allowing any binding events to be permanently ‘recorded’ through ligation of the DNA associated with both binding partners (79). An interesting feature used in bead-based DELs is enhanced stability of immobilised DNA by tethering dsDNA at both the 5′ and the 3′-end, such that even under harsh, denaturing conditions, e.g. as typically encountered during peptide synthesis, both strands of DNA remain firmly attached to the bead (74). We speculate that such an arrangement might also benefit long-term stability of the SpliMLiB DNA. A further interesting innovation in this field is the use of DNA ‘barcodes’ that—when read in combination with the synthesis-scheme-encoding DNA—render almost all beads entirely unique and thus allow discrimination during sequencing analysis between truly replicated hits and replicated hits that merely derive from PCR amplification of the DNA on a single bead (75). In our SpliMLiB Z_IgE_ campaign, the sequence diversity (160,000) was lower than the total of number of beads screened (4 million) and thus this scheme could also benefit in future from a non-protein-coding DNA barcode to help identify true replicate hits.

### Role of SpliMLiB with *in vitro* compartmentalised selections

We have shown that SpliMLiB is not only an efficient means of generating a fully non-degenerate site saturation library (which can, for example, be transformed to an expression host of choice), it can also be used in a directly screenable directed evolution platform using beads, cell-free expression mixture and compartmentalising emulsion droplets. The use of beads in droplets as clonal entities instead of cells is a well-established technique (19,20). The key advantages of the bead display approach are the avoidance of the cellular transformation bottleneck (thereby supporting large and bias-free selections *in vitro*), allowing easy delivery to the expressed protein of antigen (for protein binder selection) or of substrate (for enzyme selections), all the while exploiting the powerful features of the flow cytometer, including multiple, simultaneously operational fluorescence excitation and emission spectral bands. Our use of the SpyTag/SpyCatcher system allowed straightforward coupling of expressed protein-of-interest-SpyCatcher fusions to beads that had been modified with SpyTag, via a isopeptide bond formed between the two components (49). As both components are genetically encoded, we found the system easier to setup than the previously used SNAP display system on beads (25,27), while protein-to-bead coupling remained efficient. Due to the split & mix effect resulting in clonal clustering, we speculate that each bead should be dominated by the correct assembly, despite the indels and off-target substitutions documented by NGS analysis. Moreover, we reasoned the influence of InDels on the final screening campaign would be limited by the fact that in most cases, the C-terminally located SpyCatcher domain would have been out of frame, preventing any aberrant protein from immobilising to beads and favouring the immobilisation of full-length, intact protein in the droplet.

A significant challenge in bead display has always been achieving a sufficient amount of clonal DNA on the bead with which to program IVTT and also allowing for easy recovery of DNA after sorting (25). Notwithstanding the 8000 (80) to 30 000 (81) copies of protein that have been estimated to form from a single molecule of DNA by *in vitro* expression in a droplet, single DNA-in-droplet selections have tended to be applied in panning-type (a.k.a. ‘pull-down’) assays for protein binders (82–94), for DNA-manipulating enzymes (95–101)—where modification of the encoding nucleic acid is a powerful means of selection—and much more rarely for other enzymes (102–104). Although emulsion PCR with beads starting from single molecules of template in droplets is well-established in diagnostics (105) and in preparing for Ion-Torrent sequencing, the yield with lengths typical of proteins such as enzymes remain very low (25). SpliMLiB obviated the need for an emulsion PCR step, as the technique produces expression-ready beads coated in multiple monoclonal copies of the library variants.

Here, the utility of SpliMLiB was demonstrated through the screening at ultra-high throughput of 160 000 different Z_IgE_ variants, in a bid to affinity mature this Affibody molecule. We demonstrated robust enrichment for binding variants within the library (0 out of 48 improved variants before sorting, 17 out of 48 improved after sorting). Single mutants showed ∼2-fold improvements in binding constant, and a consensus mutant resulted in a further improvement, by up to an order of magnitude. The fact that the consensus mutant was not encountered directly in the 48 characterised hits, prompted us to return to the NGS data. It was indeed present in the SpliMLiB library, at a frequency of 9.1 × 10^−6^, and thus could have been expected to be represented by 36 physical beads in the 4 million beads sampled. We ruled out reduced protein solubility as a contributory factor to the ‘missed’ consensus sequence, as soluble, purified yield for Z_IgE_^consensus^ was 2-fold that of Z_IgE_^33^ and Z_IgE_^44^ (data not shown). We speculate that the relatively broad gate used to sort beads (ranging in Cy5 intensity from 1.7 × 10^3^ to 2.0 × 10^6^ AU) resulted in a wider distribution of binding functionalities being sorted. Future optimisation of the sorting process should thus focus on use of a narrower, more stringent sorting gate. In addition, the 48 output clones characterised here are a relatively small sample compared to other studies (e.g. the 100–200 clones characterised in a typical phage display experiment (106) or >300 clones reported to be carried out for Affibody molecules in particular (107,108)). Future implementations of SpliMLiB would benefit from characterisation of a larger number of output clones.

It is interesting to consider the limits of SpliMLiB for *in vitro* screening. The remaining bottleneck in the selection will likely be the throughput of the FACS, which at around 1400 (109)–30 000 (110) events/s (depending on desired purity and yield) limits the practical throughput to 0.5 × 10^7–^10^8^ events (assuming a 1-hour FACS session). To allow for sufficient oversampling (10-fold) to ensure complete coverage, this throughput implies a library maximum diversity of ∼10^7^ members for screening.

### Future prospects of SpliMLiB

Beyond point substitution saturation libraries, SpliMLiB will find applications in libraries that include *any* alteration: e.g. site-directed insertion or deletion libraries (111), shuffled libraries (65,112) and—on a larger scale—enzyme pathway libraries (113), by ligation of fragments that vary larger sequence motifs instead of single codons. Site-directed deletion libraries would be useful in for instance the development of genetically encoded fluorescent sensors, where deletions between domains can often result in dramatic improvements in dynamic range (114). Furthermore, we expect that developments in massively parallelised and *de novo* enzymatic synthesis of DNA (115–117) will lead to a significant drop in the price of oligonucleotides, rendering SpliMLiB ever more economical.

## DATA AVAILABILITY

Illumina MiSeq data analysed in this study has been deposited with the EBA ENA under accession number PRJEB33942.

## Supplementary Material

gkaa270_Supplemental_File

## Notes

Present address: Tuomas Huovinen, Department of Biochemistry and Food Chemistry, University of Turku, Turku, Finland.

Present address: Michael R. Snaith, Alloy Therapeutics LLC, Granta Park, Cambridge, CB21 6GP, UK.
