# Supplementary material for: Split & mix assembly of DNA libraries for ultrahigh throughput on-bead screening of functional proteins
Source: Nucleic Acids Res. 2020 May 8;48(11):e63. doi: 10.1093/nar/gkaa270 (PMC7293038; doi:10.1093/nar/gkaa270)
Supplement: gkaa270_Supplemental_File [file gkaa270_supplemental_file.pdf]

Supplementary Information for

Split & mix assembly of DNA libraries for ultrahigh throughput on-bead screening of functional proteins

Laurens Lindenburg, Tuomas Huovinen, Kayleigh van de Wiel, Michael Herger, Michael R. Snaith & Florian Hollfelder

Department of Biochemistry, University of Cambridge, 80 Tennis Ct Road, Cambridge, CB2 1GA, UK

Supplementary Figures

Figure S1. Preparation of SpyTag and azide functionalised paramagnetic microbeads. 3
Figure S2. DNA and protein sequences for $Z_{IgE}$$^{wild-type}$-SpyCatcher constructs.................................... 6
Figure S3. Comparison of DNA stability on beads,.. 10
Figure S4. Sequence-level overview of solid-phase manipulation 11
Figure S5. SpliMLiB library design for diversification of three proximal codons 12
Figure S6. Detailed sequence-level overview of SpliMLiB four-codon Z_{IgE} library 13
Figure S7. Overview of NGS analysis 13
Figure S8. Frequency of reads corresponding to truncated sequences 17
Figure S9. Validation of Z_{IgE}-IgE binding and emulsion IVTT 18
Figure S10. Proof of principle sorting of binding from non-binding Z_{IgE} 19
Figure S11. SDS-PAGE analysis for NiNTA-purified Z_{IgE}-SpyCatcher variants 21
Figure S12. BLI binding curves for selected Z_{IgE} variants 24

Supplementary Tables

Table S1. Oligonucleotides used throughout this study. 7
Table S2. Variation-encoding oligonucleotides for Z_{IgE} SpliMLiB library 8
Table S3. Codons for site saturation used in oligonucleotides for the construction of SpliMLiB Z_{IgE}..... 8
Table S4. PCR amplicons used for SpliMLiB validation and Z_{IgE} library generation. 9
Table S5. Oligonucleotide duplexes used for SpliMLiB validation and Z_{IgE} library generation. 9
Table S6. Lasers and emission filters for flow cytometer and FACS machine used in this study.......... 9
Table S7. Read copy numbers after quality filtering of MiSeq NGS data. 14
Table S8. Read counts for base, insertion or deletion 14
Table S9. Mutation counts and frequencies 16
Table S10. Sorted variants' binding signal and DNA sequences. 21
Table 11. Binding signal and DNA sequences for purified variants from input SpliMLiB library.......... 22

Supplementary Text

1. NGS analysis of SpliMLiB-generated Z_{IgE} library 24

2. Screening of SpliMLiB beads: validation of emulsion IVTT stability, Z_{IgE}-binding and functioning of flow cytometric bead sorting..27

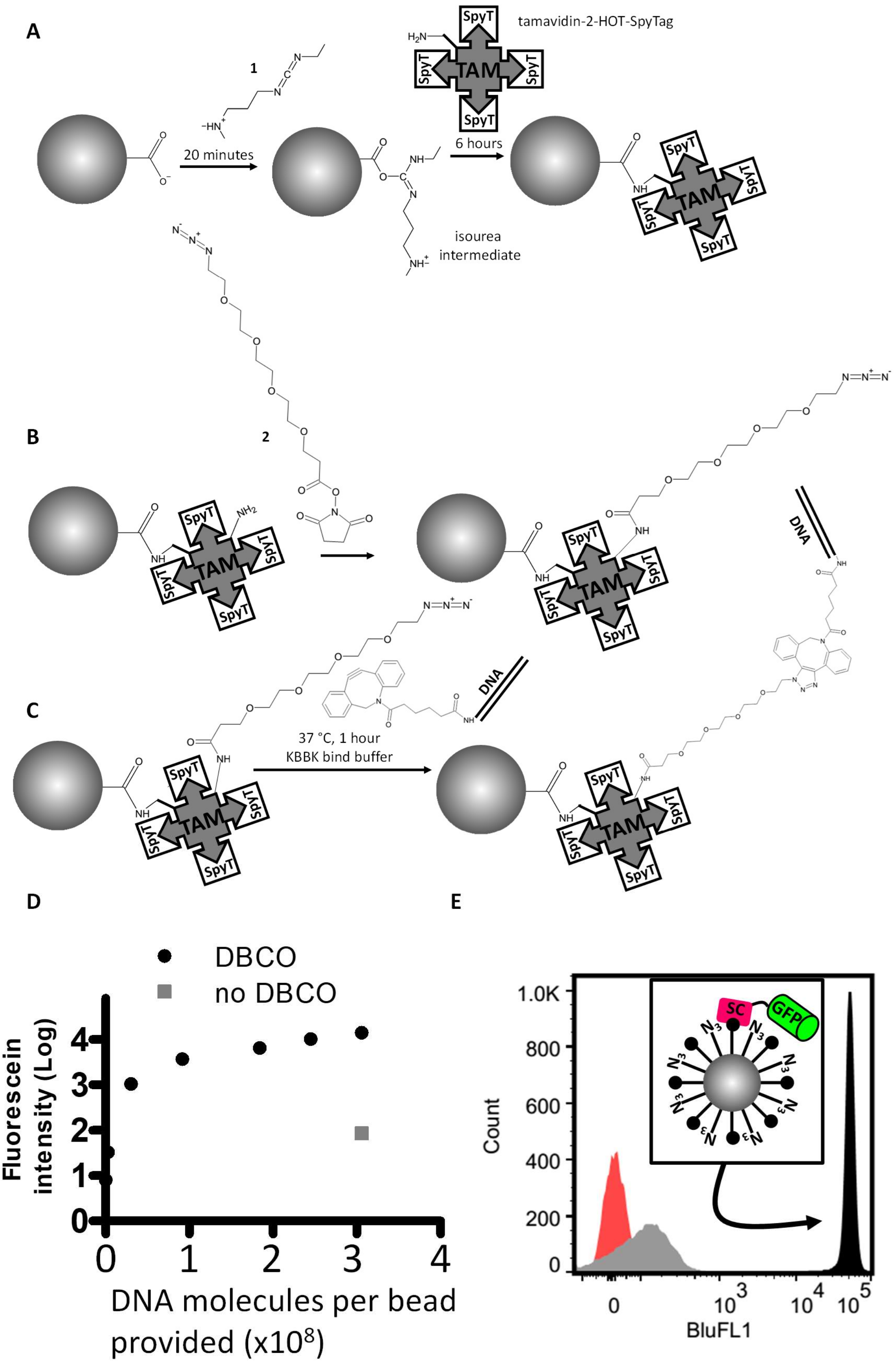

Figure S1. Preparation of SpyTag and azide functionalised paramagnetic microbeads. (**A**) The carboxylate group on paramagnetic beads was activated using EDC (1), resulting in an O-acylisourea intermediate. Subsequently, Tamavidin-2-HOT-SpyTag fusion protein was incubated with the beads,

causing the formation of an amide bond between the primary amines of lysine side chains and the carboxy functionality, with an isourea leaving group (not shown). (**B**) The beads prepared in panel (**A**) where further functionalised with an azide by incubating them with Azido-PEG4-NHS ester (2), resulting in the formation of an amide bond between remaining free lysine side chains and the activated carboxy functionality of the azide-containing molecule, with an N-hydroxysuccinimide as leaving group (not shown). (**C**) Strain-promoted azide-alkyne cycloaddition (SPAAC) reaction between the azide-functionalised beads and DBCO-functionalised double stranded DNA, resulting in the formation of a triazole linkage between bead and DNA. (**D**) Analysis of the amount of DBCO-functionalised DNA required to saturate azide-functionalised beads. A constant number of beads was exposed to increasing numbers of molecules of dsDNA (PCR.1, see Table S4) functionalised with DBCO at one end and fluorescein at the far end (black circles), under molecular crowding conditions. As a control for the specificity of the click reaction, azide-functionalised beads were exposed to a high amount of DNA (PCR.2, see Table S4) carrying fluorescein but not DBCO (grey square). (**E**) Flow cytometric analysis for SpyTag function of beads (as prepared in **C**) through exposure to either 10 µM GFP (grey distribution in frequency histogram), 10 µM GFP-SpyCatcher (black) or no GFP (red), for 1 hour in PBST at 22 °C, followed by the standard washing procedure.

A pIVEX-$Z_{IgE}^{wild\text{-}type}$-SpyCatcher map

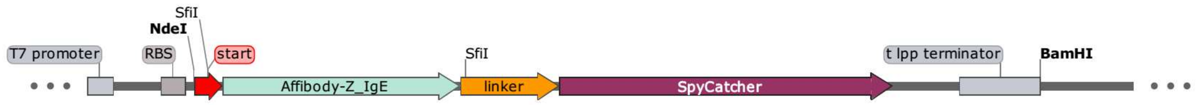

B pIVEX-$Z_{IgE}^{wild\text{-}type}$-SpyCatcher sequence (partial)

```
                                            atgtgctgcaaggcgattaagttgggt
aacgccagggttttcccagtcacgacgttgtaaaacgacggccagtgccaagcttgcatg
caaggagatggcgcccaacagtcccccggccacggggcctgccaccatacccacgccgaa
acaagcgctcatgagcccgaagtggcgagcccgatcttccccatcggtgatgtcggcgat
gaggatcgagatctcgatcccgcgaaattaatacgactcactatagggagaccacaacgg
tttccctctagaaataattttgtttaactttaagaaggagatatacatatggcccagccg
                                                    M  A  Q  P
gccatggcggtcgataacaagtttaacaaggaacccacagccgccagccttgagattatg
 A  M  A  V  D  N  K  F  N  K  E  P  T  A  A  S  L  E  I  M
atgttgcccaacctgaacgtagaccaggtaggagccttcatcgggtccctgatggatgac
 M  L  P  N  L  N  V  D  Q  V  G  A  F  I  G  S  L  M  D  D
ccttcccagtcagctaatttgctggcggaagcaaaaaagttaaacgatgcgcaagcaccg
 P  S  Q  S  A  N  L  L  A  E  A  K  K  L  N  D  A  Q  A  P
aaggcctcgggggccggaagcggtgccggtggcggaagctctggtggcggaagcggtgct
 K  A  S  G  A  G  S  G  A  G  G  G  S  S  G  G  G  S  G  A
agtgcccacctgcagtcaagcgatagtgctacccatattaaattctcaaaacgtgatgag
 S  A  H  L  Q  S  S  D  S  A  T  H  I  K  F  S  K  R  D  E
gacggcaaagagttagctggtgcaactatggagttgcgtgattcatctggtaaaactatt
 D  G  K  E  L  A  G  A  T  M  E  L  R  D  S  S  G  K  T  I
agtacatggatttcagatggacaagtgaaagatttctacctgtatccaggaaaatataca
 S  T  W  I  S  D  G  Q  V  K  D  F  Y  L  Y  P  G  K  Y  T
tttgtcgaaaccgcagcaccagacggttatgaggtagcaactgctattacctttacagtt
 F  V  E  T  A  A  P  D  G  Y  E  V  A  T  A  I  T  F  T  V
aatgagcaaggtcaggttactgtaaatggccacctgcaacatcatcaccatcaccattag
 N  E  Q  G  Q  V  T  V  N  G  H  L  Q  H  H  H  H  H  H  -
taagcttgacctgtgaagtgaaaaatggcgcacattgtgcgacatttttttgtctgccg
tttaccgctactgcgtcacggatccggctgctaacaaagcccgaaaggaagctgagttgg
```

```
ctgctgccaccgctgagcaataactagc
```

C pIVEX-CaBoFDH sequence (partial)

```
                     atgtgctgcaaggcgattaagttgggtaacgccagggttttccc
agtcacgacgttgtaaaacgacggccagtgccaagcttgcatgcaaggagatggcgccca
acagtcccccggccacggggcctgccaccatacccacgccgaaacaagcgctcatgagcc
cgaagtggcgagcccgatcttccccatcggtgatgtcggcgatataggcgccagcaaccg
cacctgtggcgccggtgatgccggccacgatgcgtccggcgtagaggatcgagatctgga
tcccgcgaaattaatacgactcactatagggagtccacaacggtttccctctagaaataa
ttttgtttaactttaagaaggagatataccatgtctggttctcatcatcatcatcatcat
agcagcggccatatgaaaattgtgctggtgctgtatgatgcaggtaaacatgcagcagat
gaagaaaaactgtatggctgcaccgaaaataaactgggtattgcaaattggctgaaagat
cagggtcatgaactgattaccaccagtgataaagaaggtgaaaccagcgaactggataaa
catattccggatgccgatattatcattaccaccccgtttcatccggcatatatcaccaaa
gaacgtctggataaagccaaaaatctgaaactggttgttgttgccggtgttggtagcgat
catattgatctggattatatcaatcagaccggcaaaaaaatcagcgttctggaagttacc
ggtagcaatgttgttagcgttgcagaacatgttgttatgaccatgctggttctggttcgc
aattttgttccggcacatgagcagattattaaccatgattgggaagttgcagccattgca
aaagatgcctatgatattgaaggtaaaaccattgcaaccattggtgcaggtcgtattggt
tatcgtgttctggaacgtctgctgccgtttaatccgaaagaactgctgtattatgattat
                                         Y   Y   D   Y
caggcactgccgaaagaagccgaagaaaaagttggtgcccgtcgtgttgaaaatattgaa
 Q   A   L   P
gaactggttgcacaggccgatattgttaccgttaatgcaccgctgcatgccggtacaaaa
ggtctgattaacaaagagctgctgagcaaattcaaaaaaggtgcatggctggttaatacc
gcacgtggtgcaatttgtgttgccgaagatgttgcagcagcactggaaagcggtcagctg
cgtggttatggtggtgatgtttggtttccgcagccagcaccgaaagatcatccgtggcgt
gatatgcgtaacaaatatggtgccggtaatgcaatgacaccgcattatagcggcaccacc
ctggatgcacagacccgttatgcagaaggcaccaaaaacattctggaaagctttttcacc
ggcaaatttgattatcgtccgcaggatattattctgctgaatggtgaatatgtgaccaaa
gcctatggcaaacatgataaaaaaggcggtggtagtgcacatattgttatggttgatgca
tacaaaccgaccaaaggtagcggtagcggccgctatccgtatgatgtaccagattatgca
agcctctaacgggatccggtaactaactaagatccggtaagatccggctgctaacaaagc
ccgaaaggaagctgagttggctgctgccaccgctgagcaataactagc
```

D pET28a-$Z_{IgE}$$^{wild\text{-}type}$-SpyCatcher sequence (partial)

```
ttttgtttaactttaagaaggagatataccatgggcagcagccatcatcatcatcatcac
                                     M   G   S   S   H   H   H   H   H   H
agcagcggcctggtgccgcgcggcagccatatggcccagccggccatggcggtcgataac
 S   S   G   L   V   P   R   G   S   H   M   A   Q   P   A   M   A   V   D   N
aagtttaacaaggaacccacagccgccagccttgagattatgatgttgcccaacctgaac
 K   F   N   K   E   P   T   A   A   S   L   E   I   M   M   L   P   N   L   N
gtagaccaggtaggagccttcatcgggtccctgatggatgacccttcccagtcagctaat
 V   D   Q   V   G   A   F   I   G   S   L   M   D   D   P   S   Q   S   A   N
ttgctggcggaagcaaaaaagttaaacgatgcgcaagcaccgaaggcctcgggggccgga
 L   L   A   E   A   K   K   L   N   D   A   Q   A   P   K   A   S   G   A   G
agcggtgccggtggcggaagctctggtggcggaagcggtgctagtgcccacctgcagtca
 S   G   A   G   G   G   S   S   G   G   G   S   G   A   S   A   H   L   Q   S
agcgatagtgctacccatattaaattctcaaaacgtgatgaggacggcaaagagttagct
 S   D   S   A   T   H   I   K   F   S   K   R   D   E   D   G   K   E   L   A
ggtgcaactatggagttgcgtgattcatctggtaaaactattagtacatggatttcagat
 G   A   T   M   E   L   R   D   S   S   G   K   T   I   S   T   W   I   S   D
ggacaagtgaaagatttctacctgtatccaggaaaatatacatttgtcgaaaccgcagca
 G   Q   V   K   D   F   Y   L   Y   P   G   K   Y   T   F   V   E   T   A   A
ccagacggttatgaggtagcaactgctattacctttacagttaatgagcaaggtcaggtt
 P   D   G   Y   E   V   A   T   A   I   T   F   T   V   N   E   Q   G   Q   V
actgtaaatggccacctgcaacatcatcaccatcaccattagtaagcttgacctgtgaag
 T   V   N   G   H   L   Q   H   H   H   H   H   H   -
tgaaaaatggcgcacattgtgcgacatttttttgtctgccgtttaccgctactgcgtca
```

```
cggatccgaattcgagctccgtcgacaagcttgcggccgcactcgagcaccaccaccacc
accactgagatccggctgctaacaaagcccgaaaggaagctgagttggctgctgccaccg
ctgagcaataactagcataaccccttggggcctctaaacgggtcttgaggggttttttgc
tgaaaggaggaactatatccggat
```

E pHAT-Avi-$Z_{IgE}^{wild-type}$-SpyCatcher sequence (partial)

```
aatacgactcactatagggaattgtgagcggataacaattcctctagaaataattttgtt
taactttaagaaggagatatatccatgaacaccattcatcaccatcaccatcacaacact
                                M   N   T   I   H   H   H   H   H   H   N   T
agtggactgaatgacattttcgaagcacagaagatcgaatggcatgaagccatggcggtc
 S   G   L   N   D   I   F   E   A   Q   K   I   E   W   H   E   A   M   A   V
gataacaagtttaacaaggaacccacagccgccagccttgagattatgatgttgcccaac
 D   N   K   F   N   K   E   P   T   A   A   S   L   E   I   M   M   L   P   N
ctgaacgtagaccaggtaggagccttcatcggtccctgatggatgacccttcccagtca
 L   N   V   D   Q   V   G   A   F   I   G   S   L   M   D   D   P   S   Q   S
gctaatttgctggcggaagcaaaaaagttaaacgatgcgcaagcaccgaaggcctcgggg
 A   N   L   L   A   E   A   K   K   L   N   D   A   Q   A   P   K   A   S   G
gccggaagcggtgccggtggcggaagctctggtggcggaagcggtgctagtgcccacctg
 A   G   S   G   A   G   G   G   S   S   G   G   G   S   G   A   S   A   H   L
cagtcaagcgatagtgctacccatattaaattctcaaaacgtgatgaggacggcaaagag
 Q   S   S   D   S   A   T   H   I   K   F   S   K   R   D   E   D   G   K   E
ttagctggtgcaactatggagttgcgtgattcatctggtaaaactattagtacatggatt
 L   A   G   A   T   M   E   L   R   D   S   S   G   K   T   I   S   T   W   I
tcagatggacaagtgaaagatttctacctgtatccaggaaaatatacatttgtcgaaacc
 S   D   G   Q   V   K   D   F   Y   L   Y   P   G   K   Y   T   F   V   E   T
gcagcaccagacggttatgaggtagcaactgctattacctttacagttaatgagcaaggt
 A   A   P   D   G   Y   E   V   A   T   A   I   T   F   T   V   N   E   Q   G
caggttactgtaaatggccacctgcaacatcatcaccatcaccattagtaagcttgacct
 Q   V   T   V   N   G   H   L   Q   H   H   H   H   H   H   -
gtgaagtgaaaaatggcgcacattgtgcgacatttttttgtctgccgtttaccgctact
gcgtcacggatccgaattcgagctccgtcgacaagcttgcggccgcatgcataagcttga
```

Figure S2. DNA and protein sequences for $Z_{IgE}^{wild-type}$-SpyCatcher constructs. (**A**) Partial plasmid map of pIVEX-$Z_{IgE}^{wild-type}$-SpyCatcher, displaying the location of T7 promoter & ribosome binding site (RBS), start codon, Z_{IgE}, linker, SpyCatcher and T7 terminator. In addition, the location of the restriction sites for NdeI, BamHI and SfiI are shown. (**B**) Partial DNA sequence of the pIVEX-$Z_{IgE}^{wild-type}$-SpyCatcher plasmid, with translation underneath the corresponding DNA sequence for the Z_{IgE}-SpyCatcher fusion protein. The NdeI (CATATG) and BamHI (GGATCC) restriction sites are underlined in the DNA sequence, the Z_{IgE} amino acid sequence is in light blue, the four targeted sites in the SpliMLiB library are highlighted in blue (T10, M18, G28 and M35), the four sites that were mutated to alanine for $Z_{IgE}^{nonbinder-1}$-SpyCatcher (T10A, M17A, V27 and G32) are underlined, the linker sequence is shown in orange, the SpyCatcher amino acid sequence is depicted in plum, while the two SfiI recognition & digestion sites (GGCCNNNNNGGCC) are highlighted in green. The LMB and T7t primer binding sites are highlighted in dark blue. (**C**) Partial DNA sequence of the pIVEX-CaBoFDH plasmid used in the SpliMLiB validation experiment for proximal codon mutagenesis. Only the DNA sequence corresponding to the one shown in the Main Manuscript (Figure 2C) is translated to amino acid sequence underneath the DNA sequence. Residues that were targeted for the 3-codon mutagenesis are highlighted in green. The LMB and T7t primer binding sites are highlighted in dark blue. (**D**) Partial DNA sequence of the pET28a-$Z_{IgE}^{wild-type}$-SpyCatcher plasmid, with translation underneath the corresponding DNA sequence for the $Z_{IgE}^{wild-type}$-SpyCatcher fusion protein. The N and C-terminal polyHis tags are italicised, Z_{IgE} is in light blue, the four sites targeted in SpliMLiB are highlighted in blue,

the linker sequence is shown in orange, the SpyCatcher sequence is in plum, the two SfiI restriction sites are highlighted in green, one NcoI recognition sites (CCATGG) is highlighted in yellow, the second NcoI site, partially overlaps with the second SfiI site and is not highlighted and the NotI recognition site (GCGGCCGC) is highlighted in red. (**E**) Partial DNA sequence of the pHAT-Avi-$Z_{IgE}^{wild-type}$-SpyCatcher plasmid, with translation underneath the corresponding DNA sequence for the Avi-tag-$Z_{IgE}^{wild-type}$-SpyCatcher fusion protein. The N and C-terminal polyHis tags are italicised, the Avi-tag sequence is in red, Z_{IgE} is in light blue, the four sites targeted in SpliMLiB are highlighted in blue, the linker sequence is shown in orange, the SpyCatcher sequence is in plum, the NcoI recognition site is highlighted in yellow and the NotI recognition site is highlighted in red.

Table S1. Oligonucleotides used throughout this study. Their use in the generation of PCR amplicons and synthetic duplexes is set out in Supplementary Table S4 and S5, respectively.

Name	5′-O-modification	purification	Sequence (5′->3′)	manufacturer
T7t_DBCO	DBCO	HPLC	GCTAGTTATTGCTCAGCGG	IDT, Leuven
T7t_biotin	biotin	desalt	GCTAGTTATTGCTCAGCGG	Sigma Aldrich, Haverhill
T7t	-	desalt	GCTAGTTATTGCTCAGCGG	
fl_end_BsmBI_F	FAM	desalt	GTATTGCGTCTCAGATGACCCTTCCCAGTCAGCTAATTTG	
before_32	-	desalt	CATCCTTAATCGCCTTGCAGCACAT	
L199W_229_F	-	desalt	GTATTGCGTCTCACAGGCATGGCCGAAAGAAGCCGAAGAAAAAGTTGGTGCCCG	
Y194A_R	-	desalt	GTTATGGCTCTTCAGTCAGCATACAGCAGTTCTTTCGGATTAAACGGC	
Y196T_F	-	desalt	GGATCCGGTGGCAAGCTGGAGGTGCTGCTCTTCAGACACC	
Y196T_R	-	desalt	CCTGGGTGTCTGAAGAGCAGCACCTCCAGCTTGCCACCGGATCC	
fl_LMB	FAM	desalt	ATGTGCTGCAAGGCGATTAAG	
TxsRed_LMB	TexasRed	HPLC	ATGTGCTGCAAGGCGATTAAG	
Unmod_LMB	-	desalt	ATGTGCTGCAAGGCGATTAAG	
SfiI_F	-	desalt	GAGATATACATATGGCCCAGCCGG	
SfiI_R	-	desalt	CTTCCGGCCCCCGAGG	
MiSeq_F	-	desalt	TCGATAACAAGTTTAACAAGGAACCC	
MiSeq_R	-	desalt	GCTGACTGGGAAGGGTCATC	

Table S2. Variation-encoding oligonucleotides for Z_{IgE} SpliMLiB library . All oligonucleotides below were from SigmaAldrich (Haverhill, England) and were synthesised without any 5'-OH modification. The Esp3I recognition sequence (CGTCTC) is in red, the 5'-single stranded overhangs that resulted from Esp3I digestion (for T10X_R and M35X_F oligonucleotides) or that were introduced by design (M18X_F & M18X-R, G28X_F & G28X_R oligonucleotides) is in blue and the mutant codon is in green. Each series consisted of 20 different oligonucleotides, varying at the position marked 'XXX', containing the site saturation codons listed in Supplementary Table S3.

Oligonucleotide series	Sequence (5'->3')
T10X_R	gttatg**CGTCTC**A**CGGC****XXX**GGGTTCCTTGTTAAACTTGTTATCGACCG
M18X_F	**GCCG**CCAGCCTTGAGATTATG**XXX**CTGCCCAAC
M18X_R	**TCAG**GTTGGGCAG**XXX**CATAATCTCAAGGCTGG
G28X_F	**CTGA**ACGTAGACCAGGTA**XXX**GCCTTCATCGGGTC
G28X_R	**CAGG**GACCCGATGAAGGC**XXX**TACCTGGTCTACGT
M35X_F	gtattg**CGTCTC**A**CCTG****XXX**GATGACCCTTCCCAGTCAGCTAATTTG

Table S3. Codons for site saturation used in oligonucleotides for the construction of SpliMLiB Z_{IgE}. The DNA sequence shown in this table was used at the position of the green 'XXX' in Supplementary Table S2 above, for oligonucleotide series 28X_F and M35X_F. For the oligonucleotide series T10X_R, M18X_R and G28X_R, the reverse complement of the DNA sequence in this table was used at the position of the green 'XXX' in Table S2.

amino acid	codon	amino acid	codon
**A**	GCT	**M**	ATG
**C**	TGT	**N**	AAT
**D**	GAT	**P**	CCG
**E**	GAA	**Q**	CAA
**F**	TTT	**R**	CGT
**G**	GGT	**S**	TCT
**H**	CAT	**T**	ACC
**I**	ATC	**V**	GTG
**K**	AAA	**W**	TGG
**L**	CTG	**Y**	TAT

Table S4. PCR amplicons used for SpliMLiB validation and Z_{IgE} library generation.

<table>
<tr><th>PCR product name</th><th>Forward primer</th><th>Reverse primer</th><th>template</th><th>Used for</th></tr>
<tr><td>PCR.0</td><td>fl_LMB</td><td>T7t_biotin</td><td rowspan="3">pIVEX-$Z_{IgE}^{wild-type}$-SpyCatcher</td><td>Fig S3A</td></tr>
<tr><td>PCR.1</td><td rowspan="2">fl_end_BsmBI_F</td><td>T7t_DBCO</td><td>Fig 2B & Fig S1D & Fig S4</td></tr>
<tr><td>PCR.2</td><td>T7t</td><td>Fig S1D</td></tr>
<tr><td>$frag_3$</td><td>L199W_229_F</td><td>T7t_DBCO</td><td rowspan="2">pIVEX-CaBoFDH</td><td rowspan="2">Fig 2C & Fig S5</td></tr>
<tr><td>$frag_1$</td><td>fl_LMB</td><td>Y194A_R</td></tr>
<tr><td>PCR.3</td><td>fl_LMB</td><td rowspan="3">T7t_DBCO</td><td>pIVEX-$Z_{IgE}^{wild-type}$-SpyCatcher</td><td>Fig 3D & Fig S3B & Fig S9</td></tr>
<tr><td>PCR.4</td><td rowspan="2">TxsRed_LMB</td><td>pIVEX-$Z_{IgE}^{nonbinder-1}$SpyCatcher</td><td>Fig S9 & Fig S10A</td></tr>
<tr><td>PCR.5</td><td rowspan="3">pIVEX-$Z_{IgE}^{wild-type}$-SpyCatcher</td><td>Fig S10A</td></tr>
<tr><td>$frag_{M35}$</td><td>M35X_F</td><td>T7t_DBCO</td><td rowspan="2">Fig 3 & Fig S6</td></tr>
<tr><td>$frag_{T10}$</td><td>fl_LMB</td><td>T10X_R</td></tr>
</table>

Table S5. Oligonucleotide duplexes used for SpliMLiB validation and Z_{IgE} library generation.

<table>
<tr><th>ON duplex</th><th>ON 1</th><th>ON 2</th><th>Used in</th></tr>
<tr><td>duplex.1</td><td>fl_LMB</td><td>before_32</td><td>Fig 2B & Fig S4</td></tr>
<tr><td>$frag_2$</td><td>Y196T_F</td><td>Y196T_R</td><td>Fig 2C & Fig S5</td></tr>
<tr><td>$frag_{G28}$</td><td>G28X_F</td><td>G28X_R</td><td rowspan="2">Fig 3 & Fig S6</td></tr>
<tr><td>$frag_{M18}$</td><td>M18X_F</td><td>M18X_R</td></tr>
</table>

Table S6. Lasers and emission filters for flow cytometer and FACS machine used in this study.

<table>
<tr><th colspan="3">FACScan Cytek</th></tr>
<tr><th>fluorophore</th><th>Excitation laser [nm]</th><th>Emission filter [nm]</th></tr>
<tr><td>Fluorescein & GFP</td><td>488</td><td>530/30</td></tr>
<tr><td>Texas Red</td><td>561</td><td>615/25</td></tr>
<tr><td>Cy5</td><td>635</td><td>666/27</td></tr>
<tr><th colspan="3">FACS Aria Fusion</th></tr>
<tr><th>fluorophore</th><th>Excitation laser [nm]</th><th>Emission filter [nm]</th></tr>
<tr><td>fluorescein</td><td>488</td><td>530/30</td></tr>
<tr><td>Texas Red</td><td>561</td><td>610/20</td></tr>
<tr><td>Cy5</td><td>640</td><td>670/30</td></tr>
</table>

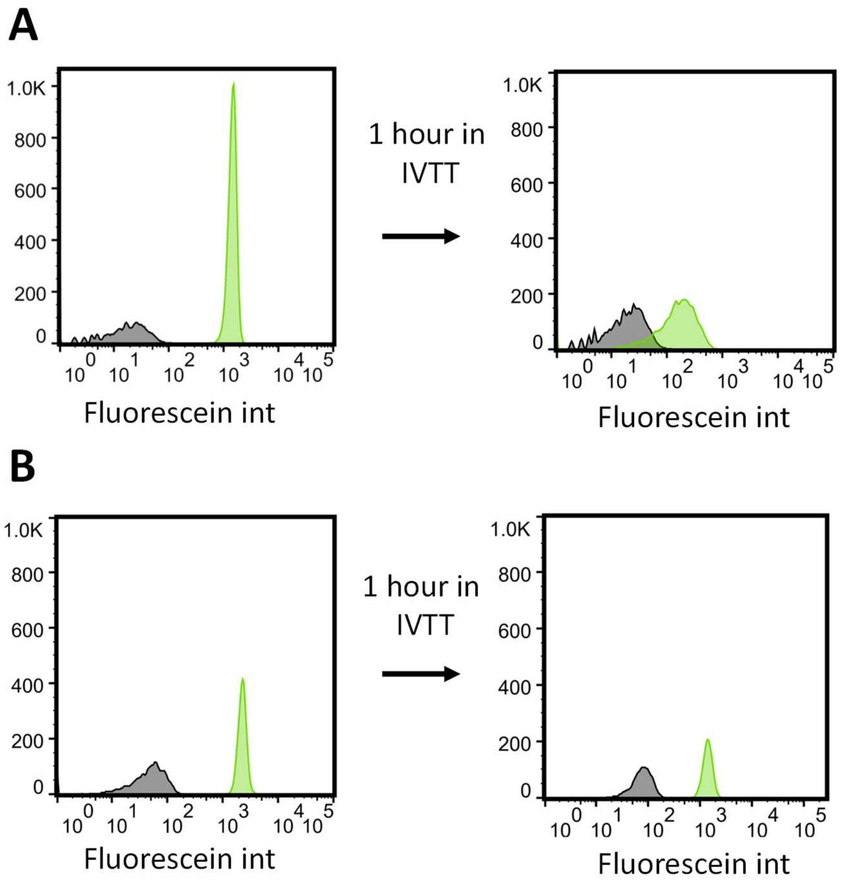

Figure S3. Comparison of DNA stability on beads, when immobilised by either biotin (**A**) or click chemistry (**B**). (**A**) Biotinylated and FAM-functionalised DNA fragment (PCR.0, see Table S4) was immobilised on Tamavidin-2-HOT-SpyTag beads (as prepared in Figure S1A) using the kilobaseBINDER Kit. These beads (green histogram), together with beads not bearing any DNA (grey histogram) were run on a flow cytometer, before (left-hand graph) and after (right-hand graph) exposure to IVTT mixture (PureXpress) for 1 hour at 37 °C. (**B**) DBCO-functionalised and FAM-functionalised DNA (PCR.3, see Table S4) was immobilised on azide-functionalised Tamavidin-2-HOT-SpyTag beads (as prepared in Figure S1B) using the kilobaseBINDER Kit (green histogram). Beads bound to DNA with a Texas Red label (grey histogram) were run on the flow cytometer as negative control for fluorescein fluorescence. The two types of bead were then mixed, exposed to IVTT for 1 hour at 37 °C and then measured again as a single flow cytometry sample (this was the same sample as shown in Figure S9). Colouring of histograms (made by gating on the well-separated fluorescein intensity) was as in the left-hand graph.

```
     Phase       immobilization PCR.1
   incoming              FAM-5'-GTATTGCGTCTCAGATGACCCTT…-3'
   solution                  3'-CATAACGCAGAGTCTACTGGGAA…-5'-DBCO--------> bead

                 Esp3I digestion
    solid-                                  P-5'-GATGACCCTT…-3'
   phase DNA                                      3'-TGGGAA…-5'-DBCO--------bead

                 ligation duplex.1
   incoming      FAM-5'-ATGTGCTGCAAGGCGATTAAG-3'
   solution          3'-TACACGACGTTCCGCTAATTCCTAC-5'-P

    solid-       FAM-5'-ATGTGCTGCAAGGCGATTAAGGATGACCCTT…-3'
   phase DNA         3'-TACACGACGTTCCGCTAATTCCTACTGGGAA…-5'-DBCO--------bead
```

Figure S4. Sequence-level overview of solid-phase manipulation (immobilisation, digestion & ligation) of DNA in Figure 2B. The Esp3I recognition site is in red, while the sites used for cohesive 5'-overhangs are in blue. A first DBCO-functionalised PCR amplicon (PCR.1, see Table S4) was immobilised to beads and then treated with Esp3I, resulting in a 5'-single stranded overhang (depicted in blue) and loss of the bead-immobilised DNA's terminal fluorescein. Next, a fluorescein-functionalised DNA duplex (duplex.1, see Table S5), carrying a 5'-phosphorylated overhang by design, complementary to the overhang of the bead-immobilised fragment's overhang, was ligated to the bead-immobilised DNA.

Step	Phase	
		immobilization frag₃
i	incoming solution	5'-GTATTGCGTCTCACAGGCATGGCCGAAAG…-3'<br>3'-CATAACGCAGAGTGTCCGTACCGGCTTTC…-5'-DBCO--------> bead
		Esp3I digestion
ii	solid-phase DNA	P-5'-CAGGCATGGCCGAAAG…-3'<br>3'-GTACCGGCTTTC…-5'-DBCO--------bead
		ligation frag₂
iii	incoming solution	5'-…AGGTGCTGCTCTTCAGACACC<br>3'-…TCCACGACGAGAAGTCTGTGGGTCC-5'-P
	solid-phase DNA	5'-…AGGTGCTGCTCTTCAGACACCCAGGCATGGCCGAAAG…-3'<br>3'-…TCCACGACGAGAAGTCTGTGGGTCCGTACCGGCTTTC…-5'-DBCO--------bead
		BspQI digestion
iv	solid-phase DNA	P-5'-GACACCCAGGCATGGCCGAAAG…-3'<br>3'-TGGGTCCGTACCGGCTTTC…-5'-DBCO--------bead
		BspQI digestion of frag₁ (off-bead operation)
n/a	incoming solution	FAM-5'-…GCTGTATGCTGACTGAAGAGCGTATTG<br>3'-…CGACATACGACTGACTTCTCGCATAAC-5'<br><br>FAM-5'-…GCTGTATGCT<br>3'-…CGACATACGACTG-5'-P
		ligation of digested frag₁
v	solid-phase DNA	FAM-5'-…GCTGTATGCTGACACCCAGGCATGGCCGAAAG…-3'<br>3'-…CGACATACGACTGTGGGTCCGTACCGGCTTTC…-5'-DBCO--------bead

Figure S5. SpliMLiB library design for diversification of three proximal codons. The three targeted positions, depicted in green, were carried by a first DBCO-functionalised PCR fragment ($frag_3$, see Table S4), a second, shorter, DNA duplex ($frag_2$, see Table S5) and a third FAM-functionalised PCR fragment ($frag_1$ see Table S4). The DBCO fragment was immobilised to beads (step i) and then treated with Esp3I (step ii), resulting in a 5'-single stranded overhang (depicted in blue). The next incoming DNA duplex carried a 5'-phosphorylated overhang by design, complementary to the overhang of the bead-immobilised fragment's overhang. After ligation of the incoming duplex (step iii), treatment with BspQI, resulted in the removal of stuffer sequence that had been added to stabilise the duplex for ligation, as well as removal of the BspQI site itself, leaving a single stranded 5'-overhang (depicted in blue). The third and final fragment was prepared off-bead with BspQI to carry a 5'-overhang and was annealed and ligated to the compatible overhang displayed on the bead.

```
  Phase    Immobilization PCR M35X
 incoming                                                            5'-GTATTGCGTCTCACCTGXXXGATGACC…-3'
 solution                                                            3'-CATAACGCAGAGTGGACXXXCTACTGG…-5'-DBCO <----> bead

           Esp31I digestion
  solid-                                                                      P-5'-CCTGXXXGATGACC…-3'
phase DNA                                                                          3'-XXXCTACTGG…-5'-DBCO--------bead

           Ligation oligonucleotide duplex G28X
 incoming                                        P-5'-CTGAACGTAGACCAGGTAXXXGCCTTCATCGGGTC-3'
 solution                                             3'-TGCATCTGGTCCATXXXCGGAAGTAGCCCAGGGAC-5'-P

  solid-                                         P-5'-CTGAACGTAGACCAGGTAXXXGCCTTCATCGGGTCCCTGXXXGATGACC…-3'
phase DNA                                             3'-TGCATCTGGTCCATXXXCGGAAGTAGCCCAGGGACXXXCTACTGG…-5'-DBCO--------bead

           Ligation oligonucleotide duplex M18X
 incoming             P-5'-GCCGCCAGCCTTGAGATTATGXXXCTGCCCAAC
 solution                  3'-GGTCGGAACTCTAATACXXXGACGGGTTGGACT

  solid-              P-5'-GCCGCCAGCCTTGAGATTATGXXXCTGCCCAACCTGAACGTAGACCAGGTAXXXGCCTTCATCGGGTCCCTGXXXGATGACC…-3'
phase DNA                  3'-GGTCGGAACTCTAATACXXXGACGGGTTGGACTTGCATCTGGTCCATXXXCGGAAGTAGCCCAGGGACXXXCTACTGG…-5'-DBCO--------bead

           Esp31I digestion of PCR T10X (off-bead operation)
           FAM-5'-…GGAACCCXXXGCCGTGAGACGCATAAC-3'
              3'-…CCTTGGGXXXCGGCACTCTGCGTATTG-5'
 incoming
 solution
           FAM-5'-…GGAACCCXXX-3'
              3'-…CCTTGGGXXXCGGC-5'-P

           Ligation of Esp31I-digested PCR T10X
  solid-   FAM-5'-…GGAACCCXXXGCCGCCAGCCTTGAGATTATGXXXCTGCCCAACCTGAACGTAGACCAGGTAXXXGCCTTCATCGGGTCCCTGXXXGATGACC…-3'
phase DNA     3'-…CCTTGGGXXXCGGCGGTCGGAACTCTAATACXXXGACGGGTTGGACTTGCATCTGGTCCATXXXCGGAAGTAGCCCAGGGACXXXCTACTGG…-5'-DBCO--------bead
```

Figure S6. Detailed sequence-level overview of SpliMLiB four-codon Z_{IgE} library (schematically depicted in main manuscript Figure 3). Mutant codons (in green) have been marked as “XXX” to reflect their saturation mutagenesis. DNA forming single-stranded overhangs for hybridisation and ligation is marked in blue. Restriction sites are marked in red.

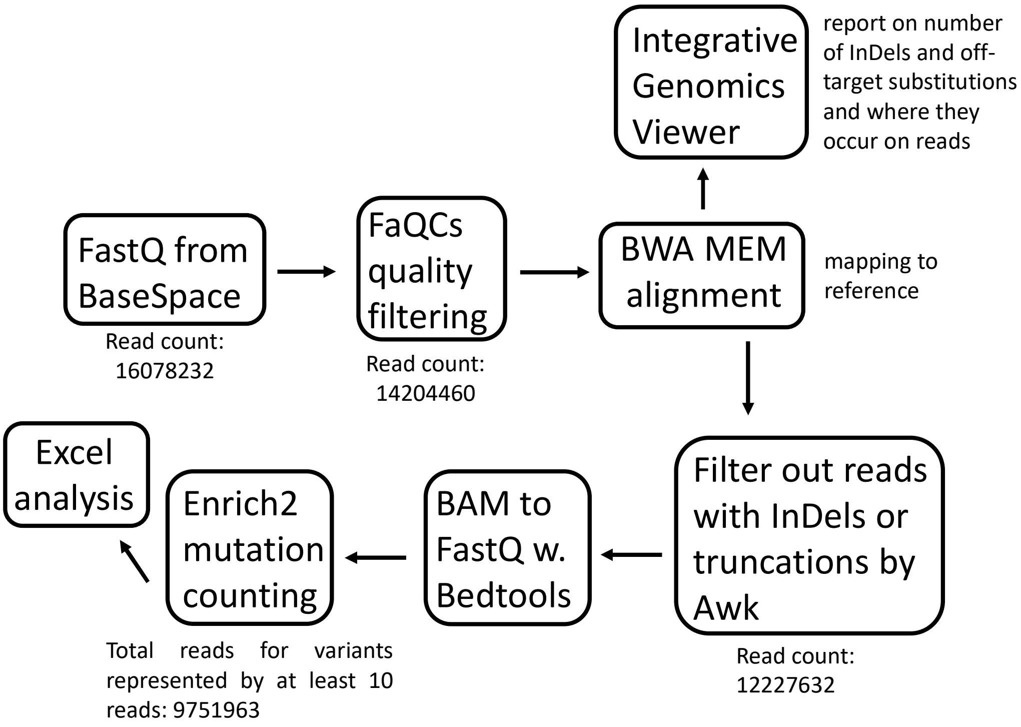

Figure S7. Overview of NGS analysis, listing the order in which data was processed and analysed.

Table S7. Read copy numbers after quality filtering of MiSeq NGS data.

Process	reads
total reads in raw fastq	16078232
discarded PhiX	1337493
discarded below average quality	530569
discarded low complexity	3
discarded reads with "N" bases	5707
Total discarded by FaQCs	1873772
Total reads after FaQCs	14204460
Total mapped by alignment (including reads with InDels)	14183030
Total after removal of reads with InDels and truncations	12227632
Enrich2 total (minimum 10 reads)	9751963

Table S8. Read counts for base, insertion or deletion occurring at each of the 78 base pairs corresponding to the sequenced SpliMLiB fragment. Only the sequence bracketed by the primers used to amplify the amplicon before TruSeq adaptor addition is shown here. Positions targeted by SpliMLiB (27-29, 51-53, 81-83 & 102-104) are indicated in red.

ref seq	bp	total	A	C	G	T	deletion	insertion
A	27	13988520	3710894	3385832	3267188	3624606	63625	5195
C	28	14020396	5614950	2393490	2936477	3075479	32132	2578
A	29	14046588	3380032	814118	3138142	6714296	9725	6986
G	30	14076603	5871	48620	13966681	55431	4734	14688
C	31	14089163	26323	14042367	17489	2984	4575	3352
C	32	14091567	12832	14070369	4389	3977	6693	145
G	33	14090126	5470	3778	14067288	13590	15423	1957
C	34	14094541	10589	14078868	2671	2413	25377	1957
C	35	14119803	21712	14090253	4318	3520	2149	38
A	36	14114235	14106138	3625	3003	1469	8816	888
G	37	14122551	2216	5872	14105884	8579	7109	2007
C	38	14123497	9824	14107827	3843	2003	13069	1449
C	39	14136712	9469	14120563	4344	2336	2300	61
T	40	14122815	2202	13872	4972	14101769	16868	736
T	41	14137545	2062	2646	3007	14129830	3120	133
G	42	14127943	3764	4017	14113806	6356	13255	767
A	43	14133473	14114395	4777	11531	2770	7937	323
G	44	14132545	3876	3135	14114832	10702	9401	524
A	45	14122024	14116480	1241	2407	1896	20038	376
T	46	14123952	1773	1584	1643	14118952	17759	626
T	47	14138228	2018	1618	1290	14133302	3282	421
A	48	14131430	14121286	2932	2970	4242	9394	474
T	49	14132137	3354	2419	6521	14119843	8038	4339
G	50	14123363	7042	50544	13973640	92137	15496	5545
A	51	14068229	3818794	3375266	3420400	3453769	46339	12048

T	52	14074010	5023339	2829168	2663683	3557820	37303	515
G	53	14080848	2131784	1463844	3272107	7213113	30923	1268
C	54	14106898	125769	13877912	54385	48832	15897	13065
T	55	14117795	1713	5758	2982	14107342	5388	6215
G	56	14116228	4592	7510	14085578	18548	8172	2855
C	57	14104910	20090	14075376	7311	2133	20484	3378
C	58	14121931	23406	14088931	6636	2958	4659	102
C	59	14126724	16028	14102402	5187	3107	1977	78
A	60	14124668	14117748	3981	1912	1027	5446	71
A	61	14127507	14117323	6076	1725	2383	4645	35
C	62	14114740	9284	14100512	3485	1459	18612	80
C	63	14127871	13616	14108849	3436	1970	5085	56
T	64	14120742	1413	2504	3689	14113136	5618	125
G	65	14118075	2767	1540	14105775	7993	8819	1878
A	66	14121783	14115033	2257	3212	1281	3405	1486
A	67	14122269	14113722	2280	4532	1735	2055	451
C	68	14116049	16161	14087898	7692	4298	6393	577
G	69	14113078	3403	3531	14095990	10154	8623	1416
T	70	14112332	5314	2472	4655	14099891	7645	385
A	71	14109026	14103259	1192	2825	1750	9554	538
G	72	14108644	3533	3972	14091146	9993	9130	1006
A	73	14108130	14098664	3012	4536	1918	6246	321
C	74	14094276	16635	14066025	8418	3198	15473	1588
C	75	14099815	19859	14071574	5202	3180	3537	59
A	76	14092715	14077236	8674	4589	2216	8471	385
G	77	14087663	2415	4029	14067084	14135	12359	1307
G	78	14092986	2872	4581	14071896	13637	3366	149
T	79	14087812	4820	5166	4237	14073589	6675	1269
A	80	14084176	14075841	2129	2967	3239	7801	1049
G	81	13993195	3465597	3429300	3386199	3712099	57684	10258
G	82	14007268	4972491	2631587	2849475	3553715	35065	909
A	83	14021562	2180691	1888197	3886594	6066080	19975	583
G	84	14033613	6164	84831	13825409	117209	9875	14256
C	85	14030306	29173	13978528	18895	3710	13011	1955
C	86	14040866	20751	14009381	7733	3001	2287	1173
T	87	14026115	2039	5391	3793	14014892	16551	859
T	88	14040120	3203	4895	1950	14030072	2208	194
C	89	14034037	15965	14011036	3997	3039	7844	1526
A	90	14034499	14026478	3508	2560	1953	6589	276
T	91	14031677	2995	4121	3451	14021110	8996	387
C	92	14026035	36056	13973023	12869	4087	9085	1647
G	93	14015501	4558	15449	13982384	13110	14498	1101
G	94	14021460	2589	3612	14001027	14232	4126	30
G	95	14017722	2546	3900	14003069	8207	3592	25
T	96	14008936	1476	3496	2697	14001267	8690	338
C	97	13985317	22964	13953202	6517	2634	26661	26829

C	98	13991980	21326	13961312	6547	2795	6532	4881
C	99	13982786	17848	13955164	5537	4237	6234	462
T	100	13970540	2370	4427	5887	13957856	14478	4463
G	101	13953798	7206	47440	13808703	90449	25801	5856
A	102	13864330	3436294	3092044	3511983	3824009	62303	15052
T	103	13871924	4778956	2884454	2648176	3560338	42235	307
G	104	13868155	1865146	1287186	4281010	6434813	44868	2207

Table S9. Mutation counts and frequencies observed at each of the four targeted positions of the SpliMLiB Z_{IgE} library.

	T10		M18		G28		M35	
mutation	count	% total	total	% total	total	% total	total	% total
A	357630	3.67%	503034	5.16%	415810	4.26%	450783	4.62%
C	634144	6.50%	518174	5.31%	521907	5.35%	422689	4.33%
D	522282	5.36%	518494	5.32%	511845	5.25%	532044	5.46%
E	550820	5.65%	462677	4.74%	453793	4.65%	453217	4.65%
F	466958	4.79%	493685	5.06%	534292	5.48%	477591	4.90%
G	454172	4.66%	498054	5.11%	435432	4.47%	526972	5.40%
H	658125	6.75%	527806	5.41%	534361	5.48%	466950	4.79%
I	553205	5.67%	497477	5.10%	470932	4.83%	399035	4.09%
K	569968	5.84%	499862	5.13%	514230	5.27%	461485	4.73%
L	328452	3.37%	438417	4.50%	464942	4.77%	507779	5.21%
M	357991	3.67%	538502	5.52%	494759	5.07%	517886	5.31%
N	515491	5.29%	568683	5.83%	520022	5.33%	498987	5.12%
P	199824	2.05%	398516	4.09%	367328	3.77%	402003	4.12%
Q	661429	6.78%	498605	5.11%	497862	5.11%	392091	4.02%
R	496587	5.09%	453844	4.65%	532203	5.46%	382919	3.93%
S	475320	4.87%	539101	5.53%	610818	6.26%	670675	6.88%
T	556065	5.70%	498445	5.11%	412613	4.23%	494813	5.07%
V	410956	4.21%	440681	4.52%	507876	5.21%	549449	5.63%
W	484020	4.96%	394417	4.04%	468634	4.81%	549015	5.63%
Y	498648	5.11%	463623	4.75%	482314	4.95%	595714	6.11%

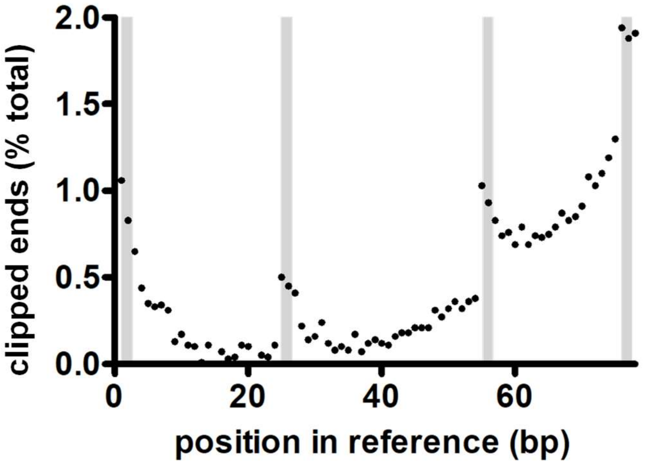

Figure S8. Frequency of reads corresponding to truncated sequences (i.e. reads aligning only to an internal fraction of the full query sequence) occurring at each position of the sequenced fragment from the SpliMLiB library. Truncations were inferred from differences in read number along the sequenced fragment in the BAM file, as analysed by IGV. The shaded bars represent the positions of the four targeted codons (from left to right, T10, M18, G28 and M35).

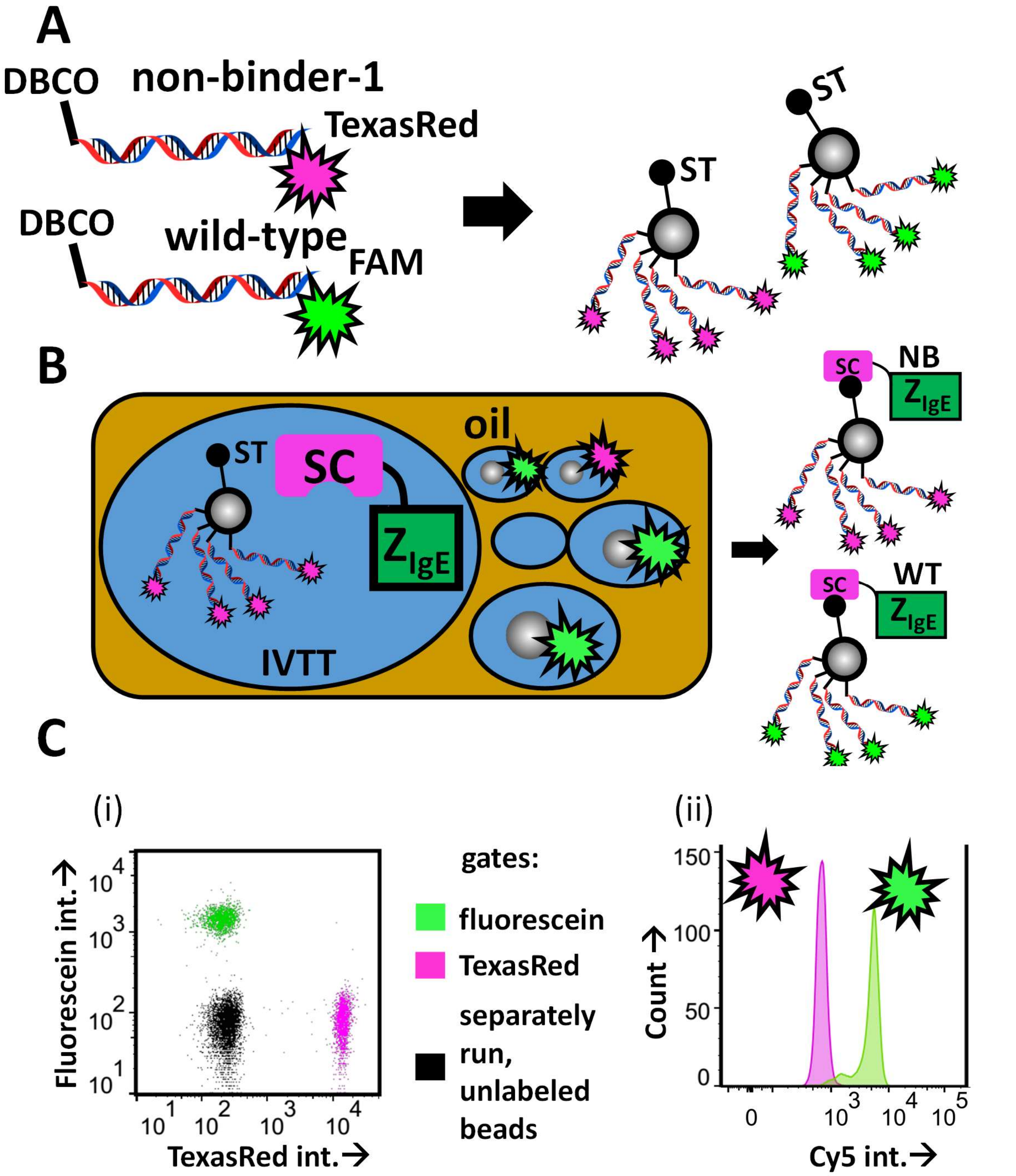

Figure S9. Validation of Z_{IgE}-IgE binding and emulsion IVTT functioning. (**A**) DNA encoding $Z_{IgE}^{non\text{-}binder\text{-}1}$–SpyCatcher (DNA labelled with Texas Red, PCR product) and $Z_{IgE}^{wild\text{-}type}$–SpyCatcher (DNA labelled with fluorescein) were immobilised on different beads and the beads mixed 1:1. SpyTag (ST) is depicted schematically. (**B**) Schematic representation of bead compartmentalisation in water-in-oil emulsion droplets containing single beads and in vitro IVTT. IVTT-expressed proteins became immobilised to beads. ST and SpyCatcher (SC) are schematically depicted. (**C**) After breaking of the emulsion, beads were stained with Cy5-labeled IgE antigen (200 nM, in 30 mg/mL skimmed milk in PBST) and analysed by flow cytometry (i). The fluorescent labels attached to the DNA allowed gating for the two different genotypes, within the IgE-binding signal (ii).

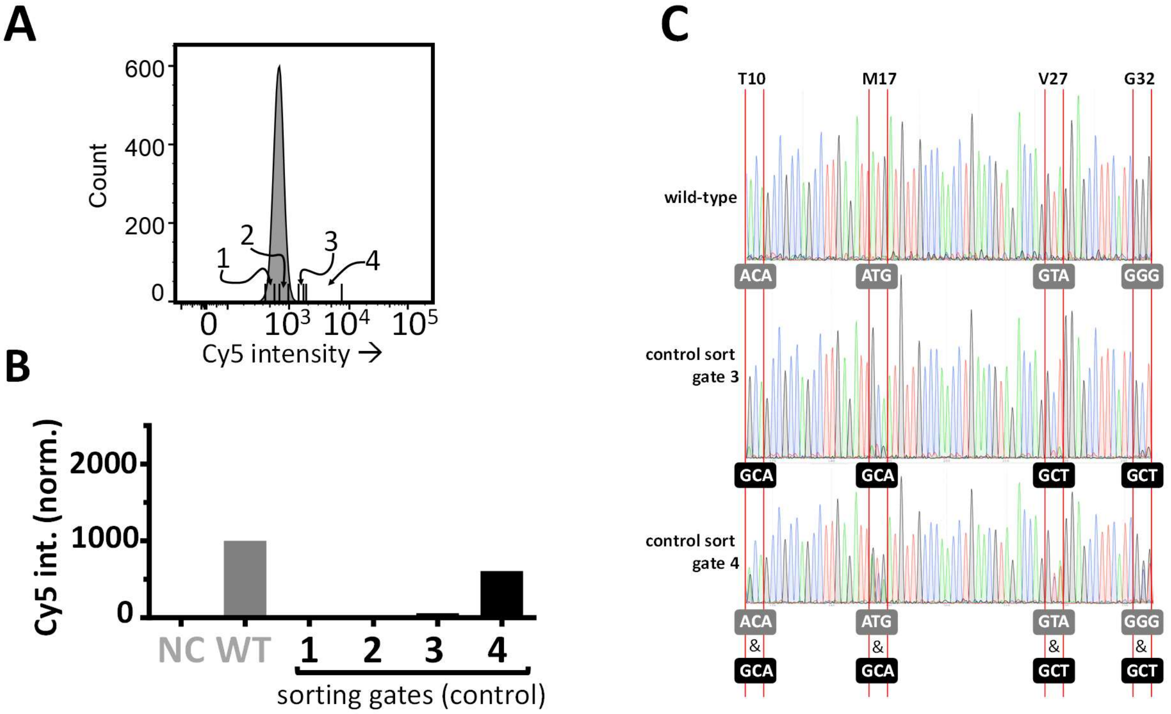

Figure S10. Proof of principle sorting of binding from non-binding Z_{IgE}. (**A**) Representative histogram recorded during the flow cytometric sorting of the control sample consisting of 0.1% beads loaded with $Z_{IgE}^{wild\text{-}type}$–SpyCatcher DNA and 99.9% beads loaded with $Z_{IgE}^{non\text{-}binder\text{-}1}$–SpyCatcher DNA. The range of fluorescence intensity used for each of the sorting gates 1-4 is indicated. (**B**) Analysis of pooled, recovered and subcloned DNA from the sorting gates set out in panel **A** for the proof-of-concept enrichment experiment. (**C**) Bulk Sanger sequencing of DNA recovered via PCR from beads sorted using the control sort gates 3 and 4 depicted in panel **A**. The Sanger sequence chromatogram of wild-type template was used as control. Sites that were mutated to alanine in $Z_{IgE}^{non\text{-}binder\text{-}1}$–SpyCatcher are labelled. While in control sort gate 3 the main peaks of the chromatogram correspond to alanine codons (i.e. $Z_{IgE}^{non\text{-}binder\text{-}1}$ genotype), in control sort gate 4 a mixture of peaks, corresponding to alanine and wild-type codons, indicates an enrichment for wild-type codons.

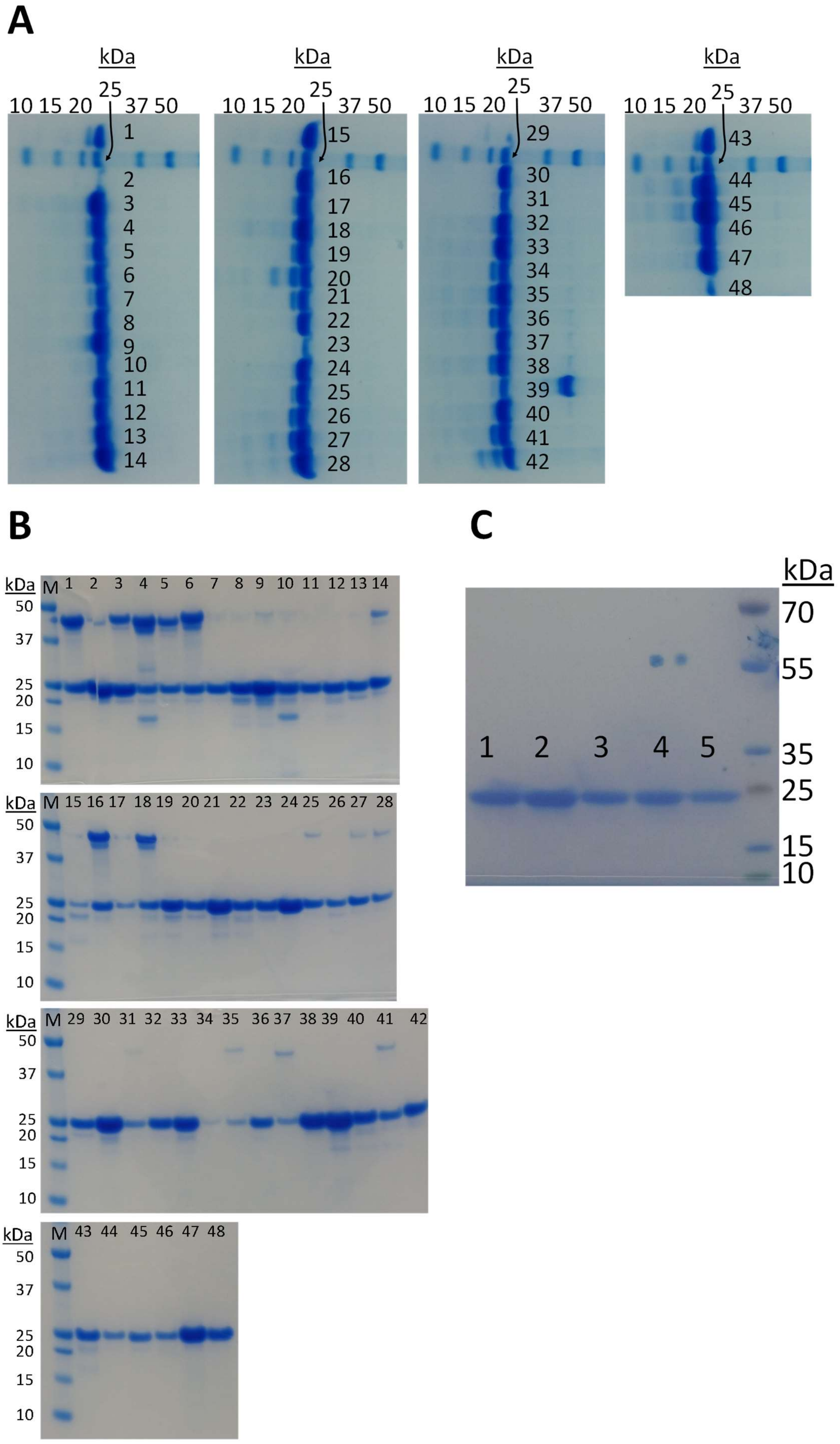
A
kDa
10 15 20 25 37 50
1 2 3 4 5 6 7 8 9 10 11 12 13 14
15 16 17 18 19 20 21 22 23 24 25 26 27 28
29 30 31 32 33 34 35 36 37 38 39 40 41 42
43 44 45 46 47 48
B
kDa
50 37 25 20 15 10
M 1 2 3 4 5 6 7 8 9 10 11 12 13 14
M 15 16 17 18 19 20 21 22 23 24 25 26 27 28
M 29 30 31 32 33 34 35 36 37 38 39 40 41 42
M 43 44 45 46 47 48
C
kDa
70 55 35 25 15 10
1 2 3 4 5

Figure S11. SDS-PAGE analysis for NiNTA-purified Z_{IgE}-SpyCatcher variants (ca 21.3 kDa). (**A**) 48 randomly selected variants from the stringent sorting gate of SpliMLiB library. The second lane (as seen from the top) in each image is the protein standards ladder (Precision Plus, BioRad). (**B**) 48 randomly selected variants from the unsorted SpliMLiB library. The first lane from left was protein standards ladder (Precision Plus, BioRad). (**C**) BirA-tagged $Z_{IgE}^{nonbinder-2}$-SpyCatcher (lane 1), Z_{IgE}^{33}-SpyCatcher (lane 2), Z_{IgE}^{44}-SpyCatcher (lane 3), $Z_{IgE}^{wild-type}$-SpyCatcher (lane 4) and $Z_{IgE}^{consensus}$-SpyCatcher (lane 5) as expressed and NiNTA-purified from 250 mL bacterial culture. The protein ladder (PageRuler Plus Prestained 10-250kDa (ThermoFisher)) was run the in right-hand lane. The higher molecular weight bands (ca 42 kDa) seen in some lanes in both (**A**) and (**B**) are correlated with cysteine-containing variants and are therefore likely to be disulphide bonded dimers (gels run under non-reducing conditions).

Table S10. Sorted variants' binding signal and DNA sequences. These bacterially expressed and purified variants were obtained from the stringent FACS sorting gate (L4) used for the Z_{IgE} SpliMLiB library. [1]The position of any deletions is indicated. [2]Off-target mutations are indicated. [3]One picked clone did not give a readable Sanger sequence.

numbering as on SDS-PAGE SI image	Cy5 intensity (normalised to WT)	sequence				notes on sequence
		T10	M18	G28	M35	
1	388.18	no sequence[3]				
2	-7.33	D	G	S	K	F30S[2]; 1-bp deletion in codon A11[1]
3	1191.61	D	S	V	A	
4	524.05	S	L	A	F	
5	902.64	N	S	S	I	
6	1151.09	S	S	S	F	
7	842.93	S	S	S	F	
8	-15.94	T	S	Q	H	
9	-7.33	Y	Q	G	E	A11V[2]
10	-11.40	E	V	Y	D	
11	1071.96	D	G	S	F	
12	1051.37	G	T	A	M	
13	518.61	N	W	A	L	
14	1181.35	D	S	V	A	
15	0.85	P	L	N	R	
16	1608.40	D	G	A	M	
17	664.43	M	S	A	L	
18	211.57	N	L	G	F	
19	-8.18	I	S	I	T	
20	-22.32	K	D	W	F	
21	622.36	G	A	G	M	

22	1111.99	D	V	A	L	
23	-16.81	S	Y	S	F	16-bp deletion (codons P20-D25)[1]
24	-22.32	G	K	S	T	
25	577.00	S	G	V	W	
26	264.56	W	M	A	M	
27	600.49	N	W	A	L	
28	1274.37	D	G	A	M	
29	-18.23	S	N	A	L	1-bp deletion in codon S13[1]
30	527.34	S	S	G	W	
31	351.16	N	L	G	F	
32	1241.65	Y	C	A	M	
33	2195.31	G	S	A	M	
34	883.52	S	M	A	I	
35	1445.48	N	S	G	M	
36	-9.98	F	Y	T	I	
37	1109.60	G	T	A	M	
38	1148.61	G	T	A	M	
39	-9.53	M	C	A	M	
40	638.30	S	S	G	W	
41	769.10	T	S	V	V	
42	14.96	Q	K	P	K	
43	1766.86	D	S	S	M	
44	1988.75	D	G	S	F	
45	1233.61	T	S	A	Y	
46	1533.04	D	G	S	L	
47	-7.26	G	K	M	T	
48	24.91	L	G	E	H	

Table 11. Binding signal and DNA sequences for purified variants from input SpliMLiB library These bacterially expressed and purified variants, from randomly picked clones from the Z_{IgE} SpliMLiB library. [1]The position of any deletions is indicated. [2]Off-target mutations are indicated. [3]One picked clone did not give a readable Sanger sequence

.

numbering as on SDS-PAGE SI image	Cy5 intensity (normalised to WT)	sequence				notes on sequence
		T10	M18	G28	M35	
1	-19.87	L	E	H	C	
2	-18.07	N	Q	R	T	
3	-17.30	H	C	N	L	
4	0.00	I	P	C	H	
5	-15.42	R	F	Q	C	

6	-70.40	C	N	V	S	
7	116.47	G	T	V	A	
8	95.92	S	W	V	D	
9	-20.73	M	D	G	D	
10	-9.59	Q	N	Q	P	
11	-21.50	T	N	H	Y	
12	-23.12	G	D	H	S	
13	932.63	S	V	A	I	
14	-42.14	N	K	K	G	
15	5.81	E	P	L	W	
16	-21.50	C	G	T	T	
17	-36.05	P	S	L	W	
18	-11.22	N	L	C	G	
19	-3.61	A	E	R	H	
20	-18.07	H	Y	V	W	
21	-26.63	I	P	A	A	
22	-9.59	V	M	T	P	
23	7.60	I	A	R	S	
24	-44.70	V	N	M	S	
25	-28.43	K	C	F	T	
26	-27.41	K	I	A	P	
27	-34.43	C	C	W	T	
28	-23.12	I	T	H	C	
29	-19.87	H	Y	V	W	
30	-35.28	H	T	L	K	36-bp deletion (codons L22-L34)[1]
31	-9.59	L	C	W	A	
32	-40.42	P	G	F	A	
33	-7.01	H	T	L	K	36-bp deletion (codons L22-L34)[1]
34	-14.64	S	D	F	P	single bp deletion in codon A29[1]
35	-24.92	C	M	S	M	
36	-6.00	D	G	T	T	G32V[2]
37	-20.73	R	I	C	M	
38	-16.44	D	H	M	V	
39	-49.76	I	P	A	A	
40	-42.99	A	P	R	A	
41	-7.01	W	P	M	C	
42	-13.02	T	A	D	N	
43	-11.99	Y	P	W	R	
44	-30.06	H	M	Y	W	
45	-23.98	L	E	Q	I	
46	-24.92	W	V	M	R	
47	-24.15	D	H	L	N	
48	-7.81	E	Q	L	T	

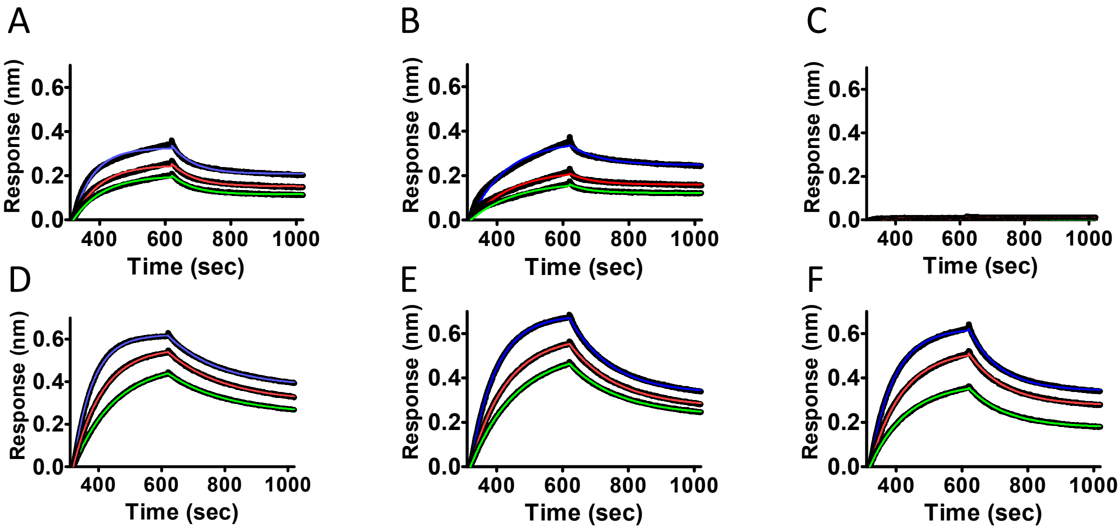

Figure S12. BLI binding curves for selected Z_{IgE} variants (with BirA tag). Data was fit to 1:1 binding model in the ForteBio Octet Data Analysis Software, assuming only partial dissociation. Blue, red and green curves are from measurements with 1, 0.5 and 0.33 µM IgE as analyte, respectively. Analysed variants were (**A**) $Z_{IgE}^{wild\text{-}type}$-SpyCatcher; (**B**) $Z_{IgE}^{nonbinder\text{-}2}$-SpyCatcher; (**C**) no Z_{IgE}-SpyCatcher control; (**D**) $Z_{IgE}^{consensus}$-SpyCatcher; (**E**) Z_{IgE}^{33}-SpyCatcher; (**F**) Z_{IgE}^{44}-SpyCatcher.

Supplementary text

1. NGS analysis of SpliMLiB-generated Z_{IgE} library

The NGS data analysis is globally described in the main Methods section (including program version numbers and references), while a schematic overview is provided in Figure S7. What follows are the command lines issued during the analysis, together with notes on the specific parameters and settings used.

To filter the raw fastq file:

```
$ FaQCs -n 1 --phiX true --avg_q 32 -u raw.fastq -d QCd_Fastq
```

"-n 1" resulted in reads possessing one or more ambiguous ("N") bases being discarded. "--phiX true" resulted in the removal of any PhiX sequences. "--avg_q 32" was included to discard reads with less than an average quality score of 32. The output ("-d") was a directory containing a file with statistics on the discarded reads (included in

Table **S7**), as well as the filtered fastq file itself. To align the filtered reads in the fastq file to the reference sequence, we first generated an index of the reference sequence:

```
$ bwa index affiref.fasta
```

This generated several files necessary for the subsequent alignment. The sequence in Affiref.fasta was:

```
>WT
```

TCGATAACAAGTTTAACAAGGAACCCACAGCCGCCAGCCTTGAGATTATGATGCTGCCCAACCTGAACGTAGACC
AGGTAGGAGCCTTCATCGGGTCCCTGATGGATGACCCTTCCCAGTCAGC

Then the BWA-MEM algorithm was run:

```
$ bwa mem -t 8 Affiref.fasta FaQCs_filtered.fastq |samtools view -h > output.bam
```

Here "-t 8" refers to the processing power to assign to the alignment. The SAM output of BWA-MEM was directly ported to SAMtools to generate a BAM file. The "-h" setting ensured the header was included in the output (necessary for the subsequent sort). Next the BAM file was sorted:

```
$ samtools sort output.bam > sorted_output.bam
```

And the sorted BAM was indexed:

```
$ samtools index sorted_output.bam
```

The sorted, indexed BAM file was analysed using IGV software. The reference sequence Affiref.fasta (see above) was loaded using Genomes → load Genome from file. The sorted BAM was then opened using File → load from file. Statistics for each bp position of the reference sequence (total reads, A, C, G, T, inserts and deletions) were copied from the consensus and used for InDel and off-target substitution analysis. This IGV output is also listed in Table S8.

Having accounted for the presence of InDels, we next proceeded to prepare for a count of library members, using only variants not containing any InDels. Reads containing InDels or that were truncated relative to the query sequence were removed by keeping only primary alignments carrying a "124M" (indicating a full-length alignment of 124 base pairs to the reference sequence) in the CIGAR tag of the BAM file:

```
$ samtools view -h sorted_output.bam \

| awk '{if($0 ~ /^@/ || $6 ~ /124M/) {print $0}}' \

| samtools view -Sb - > FullLength_sorted_output.bam
```

This is based on a slight modification of a post by the user "dariober" on the Seqanswers forum (http://seqanswers.com/forums/showthread.php?t=65696). The filtered BAM file was then converted back to fastq file format:

```
$ bedtools bamtofastq -i FullLength_sorted_output.bam -fq FullLength_sorted_output.fastq
```

Enrich2 was used to count library members and codon frequencies at each position. Its GUI was called up from the command prompt in Linux Ubuntu using:

```
$ enrich_gui
```

We started a Basic analysis for counts only. The following settings were applied, for all forward reads:

Input file: `FullLength_sorted_output.fastq` (see above)

Trim start: 27

Trim end: 78

Minimum count: 1

Wild-type sequence:

ACAGCCGCCAGCCTTGAGATTATGATGCTGCCCAACCTGAACGTAGACCAGGTAGGAGCCTTCATCGGGTCCCTG
ATG

Maximum mutations: 30

For reverse reads we used as above, except:

Trim start: 21

Wild-type sequence:

CATCAGGGACCCGATGAAGGCTCCTACCTGGTCTACGTTCAGGTTGGGCAGCATCATAATCTCAAGGCTGGCGGC
TGT

The Trim start and end settings were used to restrict Enrich2 analysis to mutations occurring within the segment of sequence enclosed by the PCR primers (MiSeq_F & MiSeq_R) used to amplify DNA prior to TruSeq adaptor addition. Mutations outside of this segment would have reflected non-SpliMLiB library related artefacts introduced by either the primers, the PCR or due to sequencing errors. The procedure was carried out twice, once for forward and once for reverse reads.

The output from Enrich2 was a list of sequences with particular mutations in DNA relative to the provided wild-type, together with a sequence copy count number. To analyse the level of representation (for potential biases and skewed distribution) of each of the 160,000 variants, we first generated a list of all theoretical SpliMLiB library members by using an Excel Macro (available from https://strugglingtoexcel.com/2014/02/21/permutations-lists-excel/). We then applied the SumIf Excel function, "=SumIf([experimental sequence range output by Enrich2], [a specific library member generated by the macro], [range for counts output by Enrich2])", so that each of the 160,000 theoretical library members became associated with a read copy count. We only counted variants associated with at least 10 reads, to ensure they were representative. In this subset of the NGS data, off-target substitutions were negligible (per position maximally 0.04%, with a median of 0.001%). The theoretical library members were then sorted in descending order of copy number.

2. Screening of SpliMLiB beads: validation of emulsion IVTT stability, Z_{IgE}-binding and functioning of flow cytometric bead sorting

Control for strict phenotype-genotype association. In the first control, we sought to confirm that the Z_{IgE}-SpyCatcher interaction with IgE could be detected reliably and that there was no cross-contamination of variants between beads during the emulsion IVTT or the subsequent bead extraction phase. To that end, separate bead populations, carrying DNA encoding $Z_{IgE}^{wild\text{-}type}$-SpyCatcher and a non-binding variant ($Z_{IgE}^{nonbinder\text{-}1}$-SpyCatcher), respectively (Supplementary Figure S9A), were mixed together and subjected to emulsion IVTT (Supplementary Figure S9B). After protein expression, the beads were extracted from the emulsion and incubated with Cy5-labeled IgE followed by analysis with flow cytometry (Supplementary Figure S9C, i). We could readily determine excellent separation of the two populations. As the two different 'genotypes' had been ear-marked with two different fluorescent labels attached to their respective DNA, this experiment suggests that the genotype-phenotype linkage was robust (Supplementary Figure S9C, ii).

Control for enrichment. In the second control, to demonstrate that the assay was suitable for sorting and that the DNA from the beads could subsequently be recovered by PCR, we carried out a proof-of-concept enrichment experiment. A mixed bead population was set up, so that 99.9% of beads carried DNA encoding the non-binding variant ($Z_{IgE}^{nonbinder\text{-}1}$-SpyCatcher) and 0.1% of beads carried $Z_{IgE}^{wild\text{-}type}$-SpyCatcher DNA. The beads were encapsulated with cell-free expression mix in droplets to express the Z_{IgE}-SpyCatcher proteins. After de-emulsification, the beads were incubated with Cy5-labeled IgE and sorted into four gates according to the Cy5 fluorescence intensity (Supplementary Figure S10A). DNA was recovered by PCR from all four different gates varying in sorting stringency (i.e. Cy5 intensity) and cloned into an acceptor plasmid (Supplementary Figure S2D). The pooled plasmid DNA from the recovery was used as template to perform IVTT (in non-emulsified, open conditions) and flow cytometry-based analysis of IgE-Cy5 binding of the expressed mixtures of Z_{IgE}-SpyCatcher was carried out. The fact that only the most stringent gate resulted in a positive binding signal (Figure S10B) clearly demonstrated the successful sorting of rare wild-type beads from the "sea" of non-binding variants. This conclusion was confirmed by sequencing a limited number of individual clones (3 out of 12 were wild-type, indicating a ~250-fold enrichment ratio) and through bulk Sanger sequencing of PCR-recovered DNA from the sorted beads (Supplementary Figure S10C).
